# Do Botanical Gardens Pose a Pollen Allergenicity Risk? A Multi-Method Assessment Integrating Species-Specific Values of Potential Allergenicity and Pollen Concentration Monitoring

**DOI:** 10.3390/plants15193063

**Published:** 2026-10-07

**Authors:** Mengxia Li, Qi Tang, Hua Zong, Xiu Li, Fangwen Li, Wenkai Chen

**Affiliations:** 1 Chengdu Botanical Garden (Chengdu Park Urban Instituted of Plant Science), Chengdu 610000, China; li94mx@163.com (M.L.); li94xiu@163.com (X.L.); m.leo@126.com (F.L.); chenwk0221@163.com (W.C.); 2 Landscape Department, Southwest Jiaotong University, Chengdu 610017, China; 13688474154@163.com

**Keywords:** botanical garden, pollen concentration, landscape trees, value of potential allergenicity, allergy risk

## Abstract

Botanical gardens serve dual functions of species conservation and public education. In these open-air recreational environments, the pollen allergy risk associated with the garden’s structure may impose considerable impacts on public health, yet this risk is often overlooked in landscape design. In this study, we conducted a comprehensive assessment of pollen allergy risk posed by landscape trees in the Chengdu Botanical Garden through landscape tree species composition surveys, flowering phenology observations, potential allergenicity values (*VPA*) estimation, and year-round pollen concentration monitoring. Six functional zones with decreasing disturbance intensity were designated in the garden. A total of 114 species (42 families, 84 genera) and 1582 individual trees were recorded, of which 76.32% are native to China. The diversity of the garden is moderately high (richness 44.8, Shannon–Wiener 3.13, evenness 0.82). A low *VPA* profile was observed in the garden. Low-*VPA* species accounted for 71.93% of species and 75.60% of individuals, while medium-*VPA* species represented 21.93% of species and 18.65% of individuals. High-*VPA* species comprised only 6.14% of species and 5.75% of individuals. Despite this low-*VPA* composition, pollen concentration remained unexpectedly high. Pollen monitoring showed that mean spring pollen concentrations in March and April reached 207.47 and 243.56 grains/cm^2^, respectively, corresponding to an extremely high risk level (Level 5). Moreover, an early peak appeared in late February (104.90 grains/cm^2^, Level 3). These elevated concentrations were primarily attributed to a highly concentrated flowering phenology, with 91.22% of landscape tree species flowering from February to May, among which 50.87% bloomed in March and April. Furthermore, sunshine duration exhibited the strongest negative association with spring pollen concentration, while mean wind speed exerted an independent positive effect after controlling for confounders, indicating that meteorological conditions significantly modulate actual exposure risk. These findings indicate that pollen allergy risk assessment should integrate both pollen concentration monitoring and tree *VPA* classification. Based on a risk framework coupling these two dimensions, the ecological conservation area (habitat protection, no public access) was identified as a high-risk core zone, the themed specialized garden area (specialist taxa display) and ecological recreation area (nature recreation, limited public access) as concentration-dominated secondary risk zones, and the educational leisure area (exhibitions and leisure), as a year-round safe zone. Given its dual mission, management should balance conservation and health through visitor guidance, physical barriers, and phenological regulation, rather than by removing allergenic species.

## 1. Introduction

In urban green space classification systems, botanical gardens are explicitly recognized as an independent and important category of urban green space, alongside urban parks, urban forests, street green spaces, and others [1]. Although botanical gardens and modern urban parks share historical origins and many urban parks have evolved from earlier botanical gardens, their contemporary functions diverge fundamentally. Urban parks, with their open spaces, shade, and aesthetic landscapes, create ideal recreational spaces and leisure opportunities for citizens [2,3]. Botanical gardens, in contrast, are defined by a more specific and mission-driven set of functions [4].

Historically, botanical gardens have often been described as living museums—cultivated spaces featuring diverse collections of plants, including rare and endangered species [5]. Their core mission has consistently centered on collection, display, research, education, and conservation. This mission-driven identity distinguishes them from general urban parks, and it is precisely this identity that has set their historical evolution in motion from private medicinal gardens to public scientific institutions. To fulfill this mission, a range of specialized activities are undertaken in botanical gardens, including maintaining seed banks and herbaria, implementing ex situ conservation programs, conducting phylogenetic and evolutionary research, monitoring climate change impacts, studying plant–animal interactions, curating garden displays, offering public services, and running educational initiatives ranging from citizen science to ethnobotany.

However, as urban expansion continues to encroach upon former suburban green spaces, this process has pushed many botanical gardens originally located on the urban fringe into increasingly central urban locations [6]. This geographic shift has prompted a rethinking and broadening of botanical gardens’ functional scope, and they are now recognized not merely as isolated repositories of plant diversity, but as key contributors to sustainable urban development [7]. In response to this changing context, relational, therapeutic, educational, and exhibition-oriented qualities that are deeply connected to their specific place-based characteristics have come to be emphasized. For example, community engagement programs, public health-oriented events, and interpretative experiences linking plant collections to residents’ daily lives may be developed, hosted, and designed.

Beyond these social functions, the ecological benefits of botanical gardens are equally remarkable. In addition to the collection of rare and endangered plants, the landscape plant diversity of botanical gardens is significantly superior to other urban green spaces in terms of species richness, community complexity, and ecosystem services. For example, the National Botanical Garden in Dhaka contained 220 tree species (59% of which were native) on a 10-hectare plot, with a Shannon–Wiener index of 1.97, far exceeding the typical index below 1.2 for ordinary urban parks [8]. Similarly, by promoting native and adaptable exotic species in landscape design, Shanghai Chenshan Botanical Garden has enriched urban horticultural plant choices, alleviated species homogenization, and achieved landscape plant diversity far surpassing that of ordinary urban green spaces [9]. This supports a wider range of wildlife and provides cross-seasonal nectar and habitat resources [10].

However, the other side of this diversity is a potential increase in pollen allergy risk. The high concentration of allergenic pollen released during flowering seasons by the diverse plant collections in botanical gardens, particularly wind-pollinated trees, may pose allergy risks to visitors, staff, and nearby residents. This risk is by no means negligible. Pollen emissions trigger allergic reactions in 30–40% of the global population, and allergic rhinitis, most commonly induced by plant pollen, affects 15–25% of people worldwide [11]. It is therefore not surprising that even in urban green spaces with lower tree diversity than botanical gardens, allergenic pollen from trees is sufficient to induce allergic symptoms [12,13]. Moreover, in urban green spaces, a key change is that pollen from landscape trees is gradually replacing herbaceous pollen as the most abundant type in urban airborne pollen [14,15]. In Spanish parks, for instance, allergenic tree species account for 17% to 67% of all species present [16].

Therefore, from a public health perspective, studying the phenological and pollen morphological characteristics of landscape trees has important practical significance. Previous studies on urban tree pollen have addressed several aspects, including the pollen morphological traits of common allergenic street trees [17], the relationship between park tree diversity and pollen emission loads [18,19], the assessment of the overall pollen allergy risk of urban parks [20,21], and investigating the spatial distribution of pollen concentrations within cities [15,22,23]. However, most of these studies have focused on ordinary parks or street green spaces, while the botanical garden, with its exceptionally high tree diversity and unique functional mission, has received little systematic attention.

By virtue of their mission to collect and conserve plant diversity, botanical gardens harbor a substantially higher richness of landscape tree species than ordinary urban parks, and they frequently include a large number of wind-pollinated taxa, with more intricate and diverse flowering phenology. Consequently, the spatiotemporal patterns of pollen allergy risk within botanical gardens are likely to be unique and cannot be directly extrapolated from findings obtained in ordinary parks. To accurately assess this risk, it is imperative to distinguish between two functionally distinct tree categories. On the one hand, rare and endangered species maintained as germplasm resources are typically limited in number and restricted to specialized conservation zones or greenhouses. These plants make a negligible contribution to airborne pollen and entail only limited visitor exposure. On the other hand, landscape trees are planted in large numbers and widely distributed across visitor-frequented areas, thus constituting the primary source of airborne pollen and the main contributors to allergy risk. This distinction is of paramount importance for any risk assessment. To elucidate the allergy risk posed by landscape trees in botanical gardens, three interrelated scientific questions merit particular attention.

First, what are the pollen morphological traits of the diverse landscape tree species in botanical gardens, and are these traits generally conducive to wind dispersal, thereby elevating the garden’s overall allergy risk? Second, how are flowering onset and duration distributed throughout the year within the species-rich landscape tree community, and is seasonal pollen exposure risk associated with this phenological distribution? Third, does the pollen concentration in the botanical garden show distinct spatiotemporal patterns, with clear peak and trough periods, and are the patterns during peak concentration periods co-regulated by meteorological factors such as temperature, humidity, wind speed, and solar radiation?

To address the above questions, this study selected Chengdu Botanical Garden as its case study and focused on landscape trees in the botanical garden. During 2023, the phenological and pollen morphological traits of all 114 landscape tree species in these areas were systematically surveyed, and year-round airborne pollen monitoring was conducted within the same zones. The spatiotemporal dynamics of pollen concentrations in these public areas were characterized, and their relationships with meteorological factors were analyzed. By integrating flowering phenology and pollen allergenic potential, targeted management recommendations were developed to inform future allergenicity-reduction strategies in botanical gardens.

## 2. Materials and Methods

### 2.1. Study Area

**Figure 1:**
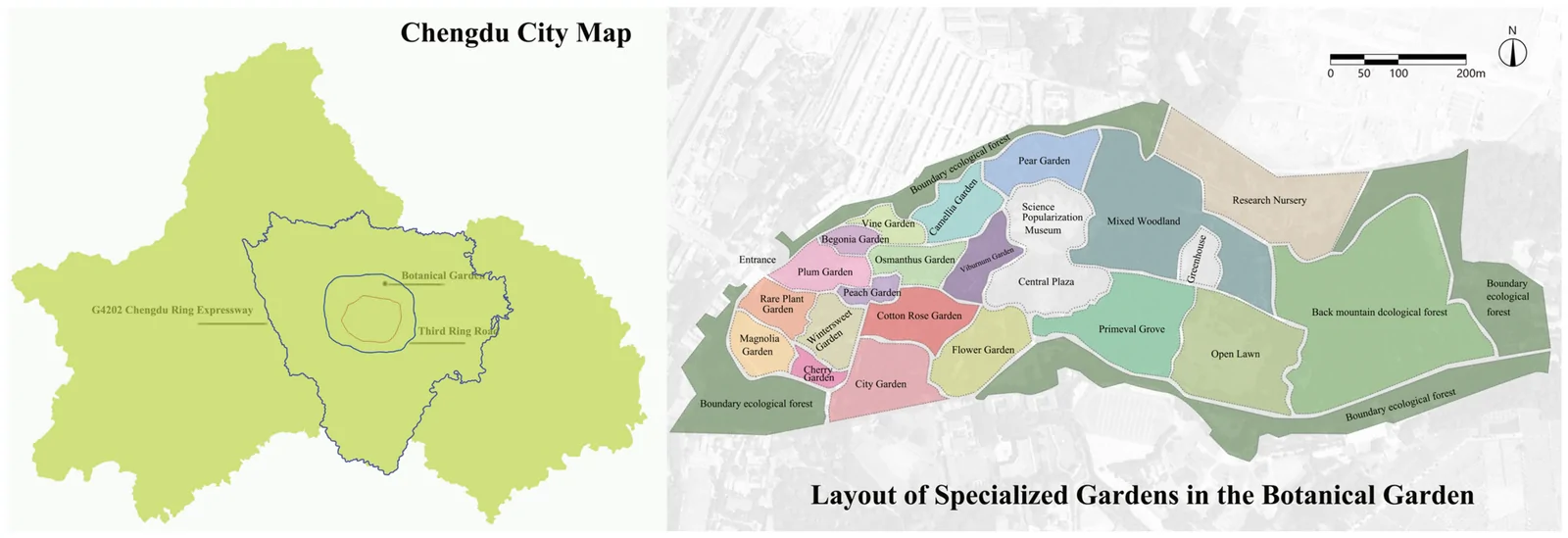
Location of the botanical garden and layout of its internal specialized gardens.

This study was conducted at the Chengdu Botanical Garden. It was established in 1983 as the first botanical garden in Sichuan Province and is located in the northern part of Chengdu city (104°10′ E, 30°40′ N). Currently, it is only 10 km from the city center, with a convenient location and easy access to transportation (Figure 1). The garden is dedicated to collecting plants from the Hengduan Mountains and the Eastern Himalayas from various altitudinal zones. In 2021, it was designated one of the 14 candidate botanical gardens under China’s national botanical garden system [24]. Since then, it has been developed into a comprehensive botanical garden that integrates multiple functions, including scientific research, public education, tourism, and recreational services.

**Figure 2:**
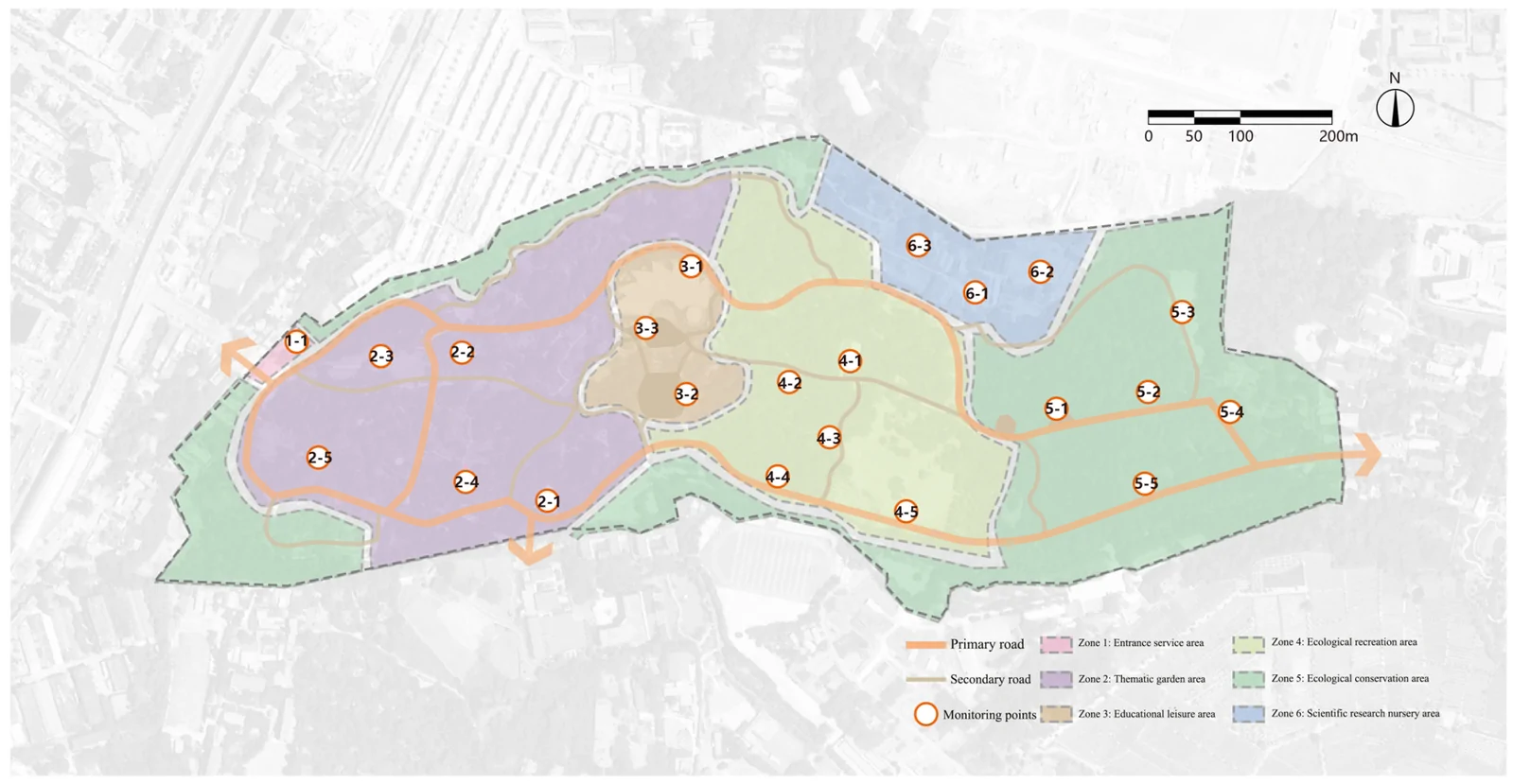
Functional zoning map of the botanical garden. Note: The numbers in the figure correspond to the pollen collection points in each functional zone.

The garden covers an area of 42.9 hectares and comprises twelve specialized plant sections (Figure 1), with a green space ratio of 94%. The elevation within the garden ranges from 496 m at the lowest point to 571 m at the highest, with considerable topographic undulation and an overall trend of higher ground to the south and east and lower ground to the north and west. Six functional management zones were designated by the botanical garden administration, forming a gradient of decreasing anthropogenic disturbance and maintenance intensity from Zone 1 to Zone 6: Zone 1 (entrance service area; visitor reception and ticketing, with high disturbance from dense visitor flows), Zone 2 (themed specialized garden area; specialist plant collection and display, with high disturbance from intensive maintenance and visitor activity), Zone 3 (educational leisure area; exhibition halls and recreational plazas, with high disturbance from public recreation and events), Zone 4 (ecological recreation area; nature-based recreation along designated trails, with moderate disturbance from limited public access), Zone 5 (ecological conservation area; habitat protection and biodiversity conservation, with low disturbance due to restricted public entry), and Zone 6 (scientific research nursery area; experimental cultivation and germplasm conservation, with very low disturbance limited to research staff) (Figure 2).

The regional climate falls within the mid-subtropical humid monsoon climate zone, characterized by mild temperatures, distinct seasons, a long frost-free period, abundant rainfall, and relatively low sunshine duration. In 2023, the total annual sunshine hours were 1239. Calm winds are the most frequent condition, with a frequency of 39%, followed by north-northeast winds (NNE), with a frequency of 14%. The multi-year annual average temperature is 16.2 °C, with an extreme maximum temperature of 37.3 °C. The total annual rainfall is concentrated primarily in July and August, with a monthly average rainfall of approximately 200 mm during these two months. The annual average relative humidity is 86%. The soil in the garden is cohesive yellow soil, with a neutral to slightly acidic pH (ranging from 6.4 to 7.2). It belongs to the Longquan Mountain system’s expansive soil, characterized by poor water retention capacity and insufficient nutrient content.

### 2.2. Data Collection and Analysis

#### 2.2.1. Recording of Landscape Tree Species and Their Diversity Characteristics

In 2022, a full-plot census was conducted to record all landscape tree species growing in the botanical garden. For the purposes of this study, landscape trees were defined as wood species growing in areas accessible to the public, where their primary function is ornamental display, shade provision, or landscape structuring. This includes: (1) roadside trees along all categories of garden paths (including internal paths within specialized thematic gardens); (2) trees within the display areas of Zones 1–4; and (3) trees along visitor-accessible routes within Zones 5 and 6. Species identification was carried out based on morphological characteristics observable in the field, including leaf shape, bark texture, inflorescence type, and fruit traits. Nomenclature followed the *Flora of China* [25]. For each recorded species, the family, genus, species, life form, and origin were subsequently compiled. The origin (e.g., native to China or exotic introduction) was determined by consulting the relevant floras [25] and historical introduction records.

To characterize the species diversity of landscape trees across the six functional zones, three *α*-diversity indices were calculated: Patrick’s richness index (*R*), Shannon–Wiener diversity index (*H′*), and Pielou’s evenness index (*J*). These three indices capture complementary dimensions of community diversity, and together they provide a more comprehensive characterization of community structure than any single index alone. By relating these indices to pollen concentration data, whether greater diversity is associated with a dilution or an accumulation effect on pollen concentration can be tested, and thus whether tree diversity can serve as a proxy for predicting allergenic risk. All indices were computed based on the species-abundance matrix for each zone using the vegan package (version 2.6.4) in R software (version 4.3.1; R Core Team, 2023).

#### 2.2.2. Flowering Phenology Observation and Pollen Morphological Data Collection

In 2023, three randomly selected 20 m^2^ plots (each containing a single species) were set up for each tree species, and at least five individuals were selected from these plots for flowering phenology observation. The onset of flowering was defined as the point at which 5% of the flowers had begun to bloom, and the end of flowering as the point at which 95% of the flowers had completely withered [26]. Observations were conducted daily in the morning. The first and last flowering dates were recorded sequentially for each species, and the average flowering duration was calculated for each species. To standardize the temporal scale, flowering dates were converted to Julian days (1 January = Day 1; 31 December = Day 365 in a non-leap year). In parallel, the experimental period was divided into seasons according to the meteorological definition, with spring spanning March to May, summer June to August, autumn September to November, and winter December to February. To facilitate the construction of a flowering phenology calendar and reduce data sparsity, the year was divided into five day bins. The Julian day intervals of each species were then mapped onto these bins, and the resulting bins were used to generate the flowering calendar.

Freshly opened anthers from ten flowers per landscape tree species were collected and air-dried. Subsequently, the anther walls were punctured with dissecting needles to release the pollen grains, which were then placed on smooth sulfuric acid paper and air-dried in the shade for 24 h before being stored for subsequent use. Pollen grains were first scanned under a light microscope (PH100-DB310U-IPL, Phenix, Shangrao, Jiangxi, China) with a 4× objective to locate pollen grains, after which they were examined and measured with a 10× objective. Exine ornamentation was further examined by scanning electron microscopy (JSM-IT500, JEOL, Akishima, Tokyo, Japan) following standard palynological protocols [27,28]. For SEM imaging, an appropriate amount of pollen was mounted onto specimen stubs using conductive adhesive tape, coated with gold in an EM ACE 200 ion sputter coater for 10 min (20 nm thickness), and then examined under a JSM-IT500 tungsten-filament scanning electron microscope at an accelerating voltage of 10 kV and a magnification of 2000×, with images captured from multiple angles.

For each species, the polar axis (*P*) and equatorial axis (*E*) lengths of 5 randomly selected pollen grains were measured. The size index (*SI*) was calculated using formula 1, and the shape was expressed as the *P/E* ratio. Exine ornamentation was categorized based on SEM micrographs. The complete morphological dataset for all 114 species is provided in Supplementary Materials Table S1.

(1) $$\mathrm{SI}=\sqrt{P\times E}$$

#### 2.2.3. Revised Estimation of Potential Allergenicity Values (*VPA*)

In landscape ecology, owing to substantial interspecific differences in flowering phenology, pollination type, and allergen levels [29], these three traits are commonly used as simple indicators to assess the potential allergenicity value (*VPA*) of plant species, and urban greening dominated by high-*VPA* species may exacerbate allergy symptoms at the neighborhood scale [16]. Cariñanos proposed the *VPA* calculation formula [16,30]:

*VPA* = *tp* × *dpp* × *ap* (2)

where *tp* is a discrete pollination type classification, with values of 1, 2, and 3 assigned to exclusively entomophilous, ambophilous (amphiphilous), and anemophilous species, respectively [16]; *dpp* is the duration of the pollination period, scored on a 1–3 scale based on flowering duration (1: 1–3 weeks; 2: 4–6 weeks; 3: ≥7 weeks) [16,30]; and *ap* is the allergenic potential of plant pollen, conventionally scored on a 0–3 scale based on literature records or medical reports (0: non-allergenicity; 1: low allergenicity; 2: medium allergenicity; 3: high allergenicity). In the present study, *ap* was classified according to the criteria established in *Mapping the Vegetation and Pollen Distribution in Northern China: Pollen Characterizations, Clinical Manifestations and Complications of Pollen Allergies* [31], which documents 43 allergenic pollen species. Species listed in this reference were categorized as highly allergenic. For taxa not included in this atlas, allergenicity values (*ap*) were determined by a systematic review of the medical literature [32,33,34]. The China National Knowledge Infrastructure database was searched for the period 1990–2025, using keywords such as “pollen allergy” and “airborne allergenic pollen.” As this database contains a large number of studies on allergen sensitization conducted in Chinese populations, it is more consistent with the physical constitution and sensitization characteristics of the Chinese population. Taxa for which pollen sensitization has been reported were classified as medium allergenicity [35], whereas those with no reported cases were classified as low allergenicity. These three indicators characterize allergenic risk from three dimensions—pollination mode, exposure duration, and allergenic intensity—and their product provides a composite reflection of the potential allergenicity of a species.

However, formula 2 still has room for further improvement. This is because the discrete *tp* classification fails to reflect the continuous gradient between entomophily and anemophily, neglects the complexity of ambophilous taxa, and lacks a quantitative metric for distinguishing the degree of anemophilous tendency among ambophilous species. To address these shortcomings, pollen morphological parameters (*SI*, *P/E* ratio, and exine ornamentation complexity) were introduced in the present study to construct a more continuous anemophily index (*Ai*), thereby providing an improved substitute for *tp*.

These three pollen morphological parameters were selected for the construction of *Ai* because they correspond to three key aerodynamic dimensions—pollen size, shape, and surface ornamentation—and have been widely confirmed to be significantly associated with wind dispersal efficiency, making them reliable indicators of pollination mode. As previously reported, anemophilous pollen is generally smaller, smoother, and thinner walled [36,37], and is often spherical with fewer apertures [38,39], whereas entomophilous pollen tends to exhibit more complex ornamentation [40]. These traits influence the aerodynamic properties and dispersal distance of pollen [39], and even spine spacing may be subject to pollinator-mediated selection [41]. In contrast to the original *tp*, which relies solely on the literature to make a discrete three-category classification of pollination mode, *Ai* converts the categorical assignment of entomophily–ambophily–anemophily into a continuous measure along an anemophily gradient through continuous scoring based on these three quantifiable traits, thereby characterizing the differences in anemophilous tendency among ambophilous taxa more precisely and avoiding the information loss inherent in discrete classification.

In formula 2, *dpp* is scored on a 1–3 scale, whereas *ap* is scored on a 0–3 scale to distinguish non-allergenicity from different grades of allergenicity. To maintain consistency in scoring range with these two components, *Ai* was also scored on a 1–3 scale. Each parameter used to calculate *Ai* was scored from 1 to 3 according to its aerodynamic suitability for wind dispersal (Table 1). The class boundaries for each trait were determined based on previously published studies of pollen morphology, and the scoring direction was defined according to the typical morphological characteristics of anemophilous pollen such that trait states more closely matching the anemophilous syndrome were assigned the highest score 3, whereas those deviating more strongly were assigned the lowest scores 1.

Specifically, the scoring of *SI* was based on previous reports [42,43], in which anemophilous pollen grains were reported to be mostly concentrated in the range of 20–50 μm; therefore, this range was assigned a score of 3, whereas 10–20 μm and 51–70 μm, which deviate from the typical anemophilous range, but remain within the medium size class, were assigned a score of 2, and values below 10 μm or above 70 μm, which clearly deviate from anemophilous characteristics, were assigned a score of 1. The class boundaries for *P/E* were based on the report of Erdtman [27], in which near-spherical pollen grains within the range 0.88 < *P/E* < 1.14 were indicated to have the lowest aerodynamic resistance and thus the greatest advantage for wind dispersal, and were therefore assigned a score of 3; grains with 0.5 ≤ *P/E* ≤ 0.88 or 1.14 ≤ *P/E* ≤ 2 were assigned a score of 2; and grains with *P/E* < 0.5 or *P/E* > 2, which are least favorable for wind dispersal, were assigned a score of 1. The class boundaries for exine ornamentation were based on the report of Tao and Sannier [44,45], in which smooth or simply ornamented surfaces (psilate, granular, or verrucate-rugulate) were shown to have relatively low aerodynamic resistance and were therefore assigned a score of 3; moderately complex types (striate, foveolate, or cerebroid) were assigned a score of 2; and complex types with prominent protrusions (reticulate, echinate, tuberculate, or verrucate) were assigned a score of 1.

It should be noted that the *Ai* scoring system was developed specifically for the present study as an improvement on the original *tp*, rather than being a previously validated index. Its scoring criteria were established based on published relationships between pollen morphology and pollination mode. Because the primary aim of this study was not to develop a general-purpose pollination-mode discriminator but to provide a more continuous and discriminative substitute for *tp* in VPA calculation, no separate sensitivity analysis or external validation was performed. This methodological choice may introduce some uncertainty, and future studies could further test the robustness of the index by comparing it with known pollination modes, expert scoring, or perturbation analysis. Finally, VPA was subsequently calculated using formula 3:

*VPA* = *Ai* × *dpp* × *ap* (3)

*VPA* values were then classified into three allergy levels: low (*VPA* ≤ 6), medium (6 < *VPA* ≤ 16), and high (*VP* > 16) [16,30].

#### 2.2.4. Monitoring of Seasonal Pollen Concentrations in Different Areas

**Figure 3:**
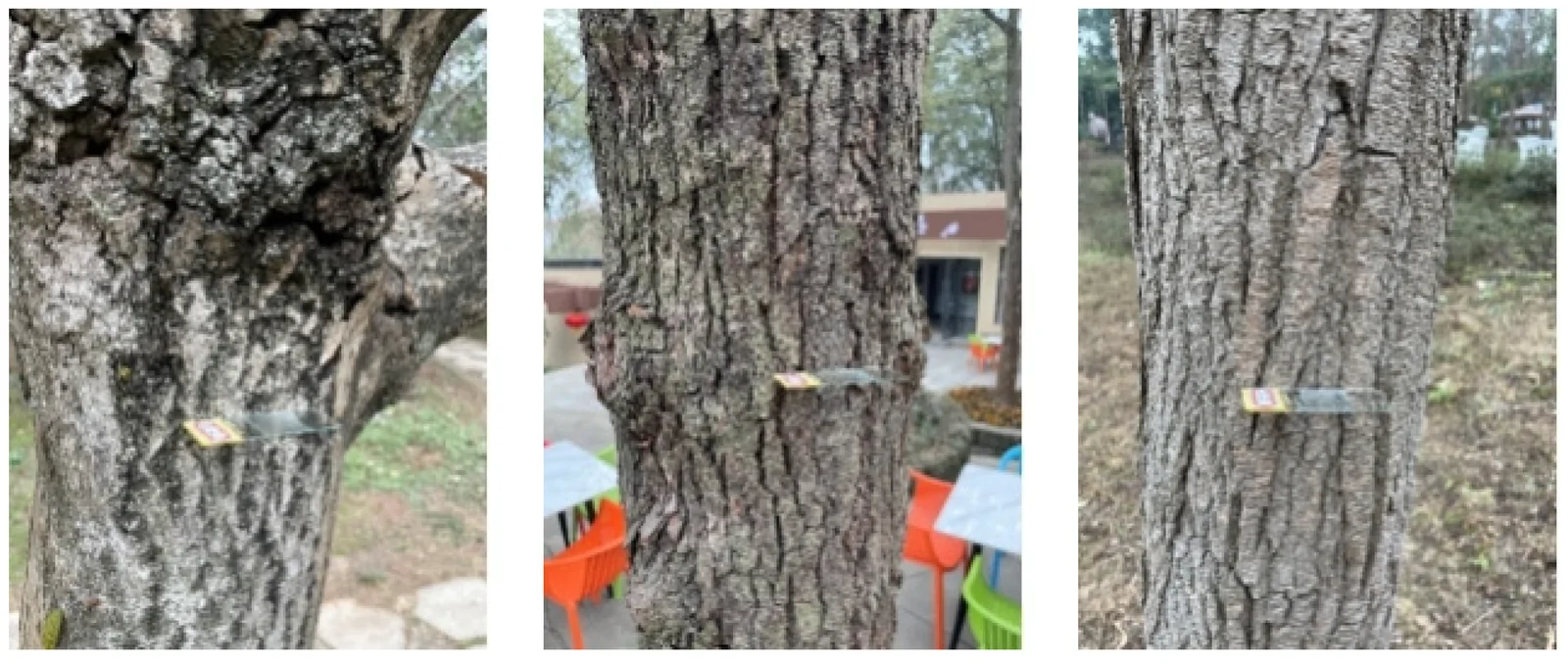
Placement diagram of the tree-suspended slide sampling device.

To align the pollen concentration risk assessment with the existing operational framework of the botanical garden, we restricted the deployment of monitoring sites to areas where landscape tree species are the primary focus. Specifically, sites were established along the various garden paths that connect the different functional zones and serve recreational purposes. After minor local adjustments (e.g., to ensure vegetation homogeneity), a total of 22 monitoring sites were ultimately deployed throughout the garden (Figure 2 and Figure 3). The mean pollen value reported here represents the total pollen assemblage collected at each site, i.e., it includes all pollen taxa; however, because all sites were located in areas where landscape trees dominate, the assemblage is expected to be dominated by landscape tree pollen.

Pollen grains were collected using the gravity sedimentation method. A thin, uniform layer of Vaseline was applied to the center of each glass slide to act as an adhesive for trapping pollen grains. Pollen abundance is therefore expressed as the number of pollen grains deposited per unit area, i.e., grains per cm^2^. A label was affixed to the left side of each slide, indicating the sampling date and location to facilitate subsequent data recording. To reflect human exposure and support allergy risk assessment, the slides were positioned at a height of 1.5 m, corresponding to the average human breathing zone. At each monitoring site, the slides were held in slots created by two parallel nails driven into tree trunks at the same height, ensuring they remained secure and preventing loss or displacement. Three replicate slides were deployed at each site.

Based on preliminary monitoring results (spring 2021–spring 2022), peak pollen occurred in March–April, moderate levels in February and May, and low levels in the remaining months. Therefore, a variable sampling frequency was adopted for the formal monitoring program (December 2022–December 2023): 8 days/month in March–April (equivalent to approximately twice per 7-day interval), 5 days/month in February and May, and 2–4 days/month in the remaining months. In spring, sampling was most intensive, with two days per 7-day interval in March–April to better capture short-lived peak pollen events, and once every 7 days in May. In summer, the sampling frequency was reduced, with sampling once every 7 days in June, once every 8 days in July, and approximately once every 10 days in August. In autumn, sampling was conducted approximately once every 10 days. In winter, sampling was conducted approximately once every 12 days from December to January, and once every 6 days in February. This variable-frequency design was intended to match sampling effort to expected pollen abundance, thereby ensuring that periods of maximum pollen release were sampled most intensively while avoiding unnecessary sampling during low-pollen months. In the one-year airborne pollen concentration monitoring experiment, 45 days of monitoring results were finally obtained, distributed across the seasons as follows: 21 days in spring, 8 days in summer, 6 days in autumn, and 10 days in winter. Slides were exposed for 24 h (10:00 a.m. to 10:00 a.m. the next day) per sampling day, and sampling was carried out only on days without precipitation to avoid the washout effect of rainfall on airborne pollen deposition.

Given that the main pollen peaks in this region typically persist for several days to one week, a frequency of two days per 7-day interval during the peak period was sufficient to capture short-lived peak events, whereas the lower frequency in the remaining months was adequate because pollen concentrations were consistently low. Although the risk of missing short-lived peak events was minimized, the possibility that some peak events were not captured cannot be entirely excluded, and the assignment of concentration levels may therefore have been slightly affected.

The retrieved slides were placed flat on the laboratory bench. A drop of carbol–fuchsin stain was first applied, followed by covering with a 20 mm × 20 mm coverslip. After staining for 5–10 min at room temperature, the slides were examined under a light microscope. The stained area was initially scanned at 4× magnification to locate pollen grains, and then examined at 10× for detailed observation and photomicrography. For each slide, the morphology and number of pollen grains were carefully recorded. The pollen count within a 4-cm^2^ area was then converted to the number per cm^2^ to give the pollen concentration (grains/cm^2)^. After averaging the counts from the three replicate slides, the mean concentration was assigned to one of five concentration levels, following the index of pollen allergy from the China Meteorological Service Center [46] (Table 2). This criteria reflects airborne pollen exposure intensity, not individual clinical allergy risk. Seasonal means of pollen concentration were then calculated as weighted averages based on the number of sampling days per month, rather than as simple averages of the monthly means.

#### 2.2.5. Climate Data Collection

During the pollen concentration observation period, meteorological data including air temperature, relative humidity, wind speed, and sunshine duration were continuously recorded by a Visala Milos automatic weather station (Vaisala Oyj, Helsinki, Finland) installed on the terrace of the botanical garden’s science education building. Data were logged every three minutes, and subsequently compiled into a master table in Excel 2016 for further analysis.

#### 2.2.6. Statistical Analysis Methods

Descriptive statistics were first computed for the morphological traits of landscape tree pollen, and the mean, standard deviation, and coefficient of variation were calculated. To compare spring pollen concentrations among the six functional zones, the normality and homogeneity of variances were first assessed using the Shapiro–Wilk test and Levene’s test, respectively. If the assumptions were violated (*p* < 0.05), the non-parametric Kruskal–Wallis test was used for multi-group comparisons, followed by Dunn’s post hoc test with Bonferroni correction to control the family-wise error rate when the Kruskal–Wallis test was significant (*p* < 0.05).

Kriging interpolation was then performed using ArcGIS 10.8 software based on the concentration data from 22 monitoring points to generate spatial distribution maps of pollen concentration for each season. Spearman correlation analyses were performed both at the whole-garden scale and separately for each of the six functional zones, in order to examine the associations between individual meteorological variables and pollen concentrations in different zones at different spatial scales. A generalized linear model (GLM) was then constructed at the whole-garden scale to further identify the independent effects of meteorological variables after controlling for confounding factors. Data were organized in Excel 2016 software, statistical analyses were conducted in SPSS 11.0 (SPSS Inc., Chicago, IL, USA), and graphs were plotted using Origin 8.0 (OriginLab Corporation, Northampton, MA, USA) and ArcGIS 10.8 software (ESRI Inc., Redlands, CA, US, USA).

## 3. Results

### 3.1. Composition and Origin Characteristics of Landscape Trees

**Figure 4:**
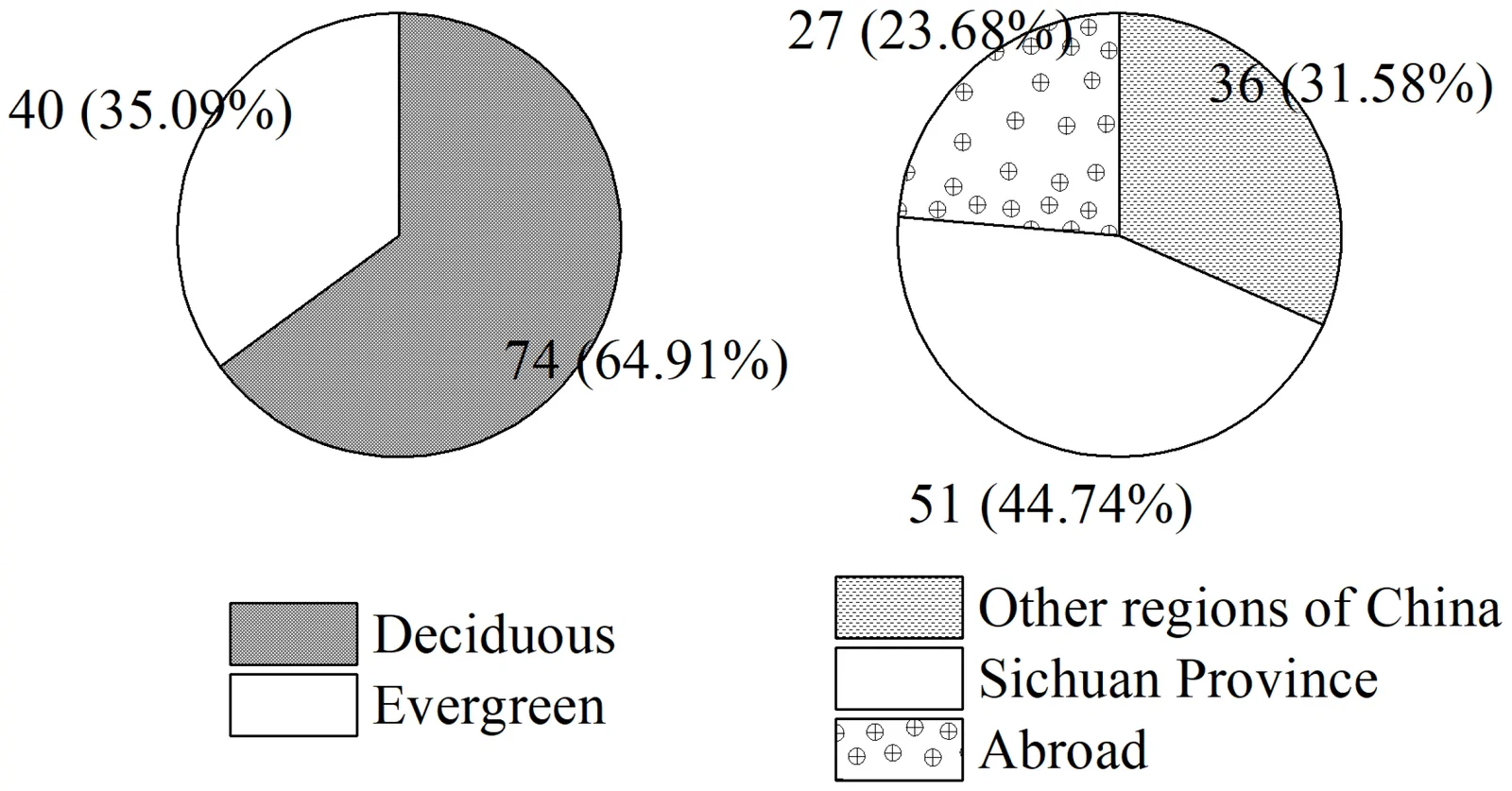
The life form and origin of tree species in the botanical garden.

The characteristics of landscape tree species in the botanical garden are summarized in Appendix A. A total of 114 landscape tree species were identified, representing 1582 individual trees and belonging to 42 families and 84 genera. Rosaceae was the most species-rich family, comprising five genera and 19 species, accounting for 16.67% of the total tree species. Of these 19 species, 15 belong to the genus *Prunus*, which was the most species-rich genus. Fabaceae followed, with six genera and eight species, and Magnoliaceae with four genera and eight species, each representing 7.02% of the total species. The remaining 21 families each contained only one genus and one species, including Ginkgoaceae, Platanaceae, Proteaceae, and Hamamelidaceae. In terms of growth habit (Figure 4), evergreen species predominated, with 74 species (64.91%) compared to 40 deciduous species (35.09%). With regard to origin, 87 tree species are native to China, representing 76.32% of the total species. Of these, 51 species are locally native to Sichuan Province, accounting for 44.74% of the total species. Notably, among the native landscape plants are six species of national key protected tree, including *Davidia involucrata*, *Sinojackia xylocarpa*, and *Magnolia zenii*, among others.

### 3.2. Flowering Phenology of Landscape Trees

**Figure 5:**
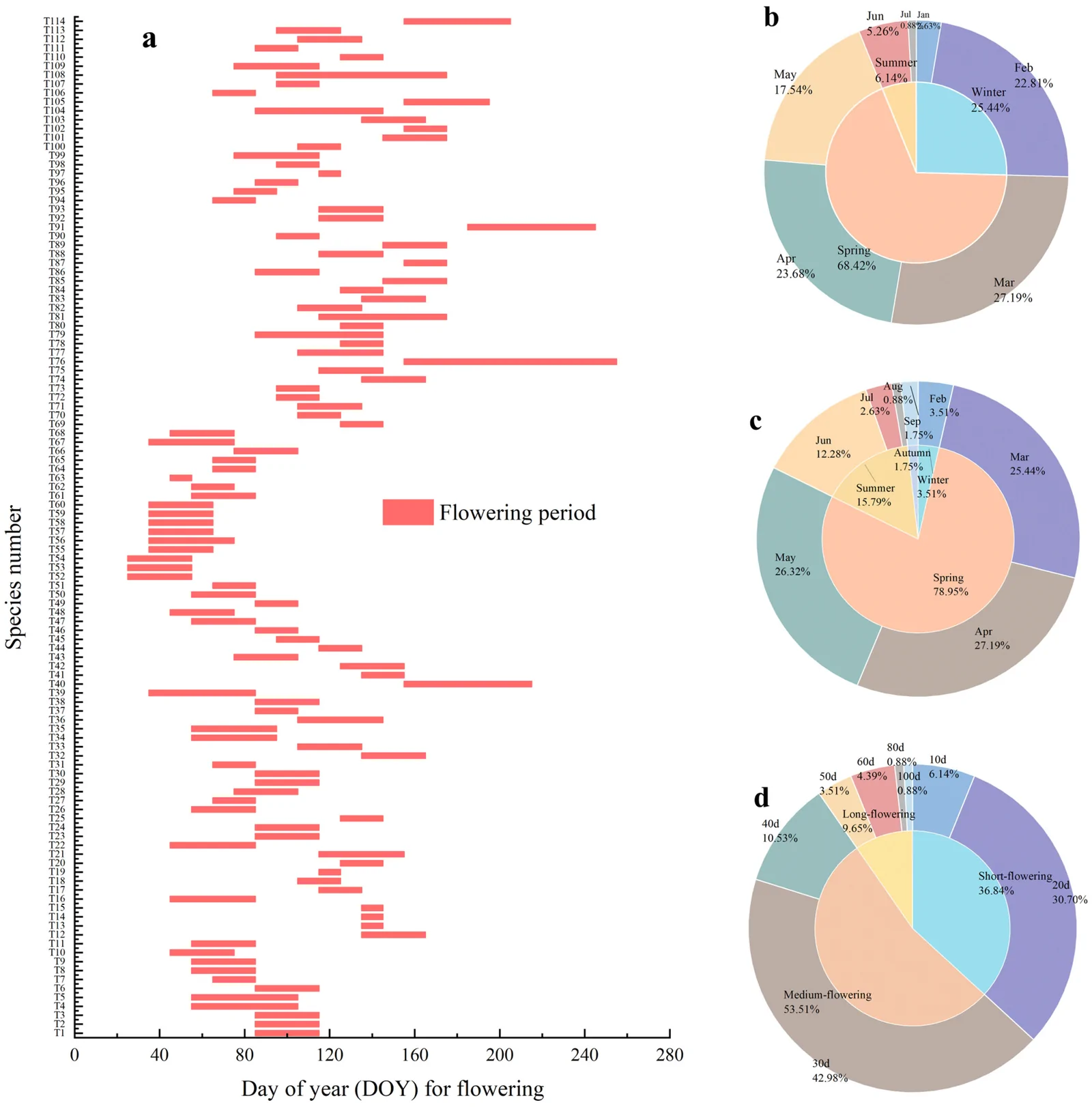
Flowering calendar of landscape trees. (**a**) Start and end dates and flowering duration of individual landscape tree species; (**b**) seasonal composition of *FFD*; (**c**) seasonal composition of *EFD*; (**d**) proportional distribution of flowering duration categories (short, medium, long).

As shown in Figure 5a, the Julian day of the first flowering date (*FFD*) of the 114 landscape tree species ranged from 25 to 185, corresponding to the period from late January through early July and spanning winter, spring, and summer. The earliest *FFD* occurred in late January, observed in *Prunus mume* cultivars including “Zi Di Bai” and “Bian Lü-e,” as well as *P. mume* var. bungo. *Koelreuteria paniculata* had the latest *FFD*, beginning to flower in early July. Preceding it were *Firmiana simplex*, *Lagerstroemia indica*, *Euonymus maackii*, *Toona sinensis*, *Vitex negundo* var. *cannabifolia*, and *Crateva formosensis*, all of which had their *FFD* in early June. A family-based analysis of *FFD* revealed that Theaceae had the earliest average *FFD*, occurring in late winter (early February). This was followed by Rosaceae, with an average *FFD* of approximately Julian day 47.11, corresponding to around 17–18 February. Celastraceae, Verbenaceae, and Capparaceae had the latest mean *FFD*, all occurring in early June (summer).

At the seasonal level (Figure 5b), the majority of tree species (78, 68.42%) initiated flowering in spring. The proportion of species with winter-onset flowering was 25.44%, while summer-onset species were relatively rare, accounting for only 6.14%. At the monthly level, the proportions followed the order: March (27.19%) > April (23.68%) > February (22.81%) > May (17.54%) > June (5.26%) > January (2.63%) > July (0.88%). This distribution clearly demonstrates that first flowering dates (*FFD*) are highly concentrated from late winter (February) to spring (March to May), during which period 91.22% of all species began flowering, and of these, 50.87% bloomed in March and April. In contrast, less than 9% of species flowered under extreme conditions, such as low temperatures (January) or high temperatures (June to July).

For the end of flowering date (*EFD*), the Julian day across 114 species ranged from 55 to 255. The earliest *EFD* also occurred in late February, corresponding to the early-flowering species. Among the species examined, *Koelreuteria paniculata* and *Lagerstroemia indica* had the latest *EFD*, occurring in early and mid-September, respectively. At the family level, Rosaceae had the earliest mean *EFD* (Julian day 74, around 13–14 March), whereas Capparaceae had the latest mean *EFD* (approximately late July) and Lythraceae also had a relatively late mean *EFD* (around mid-July).

Figure 5c presents the proportional distribution of *EFD* among the 114 species across seasons and months (February to September). The *EFD* values were concentrated in spring, accounting for 78.95%, of which March, April, and May represented 25.44%, 27.19%, and 26.32%, respectively. The proportions for spring months were relatively balanced, suggesting that the peak flowering period extended continuously throughout the spring. A total of 15.79% of species had their *EFD* in summer, while only 5.26% had *EFD* extending into autumn and winter combined.

Based on the flowering duration, trees species were classified into three categories: short (≤20 days), medium (21–40 days), and long (>40 days). Figure 5d shows that species with a medium flowering duration were the most abundant, accounting for 53.51%, with durations mostly concentrated around 30 days. Short-flowering species accounted for 36.84%, with 20 days being relatively common. Only 9.65% had a long flowering duration, lasting more than 40 days. At the species level, the shortest flowering duration (only 10 days) was observed in seven species: *Cinnamomum camphora*, *Magnolia delavayi*, *Magnolia zenii*, *Manglietia grandis*, *Prunus pseudocerasus*, *Toona sinensis,* and *Toxicodendron vernicifluum*. *Lagerstroemia indica* had the longest duration, lasting 100 days, followed by *Ligustrum lucidum* (80 days).

### 3.3. The Anemophily Index (Ai) and Potential Allergenicity Values (VPA) of Landscape Trees

**Figure 6:**
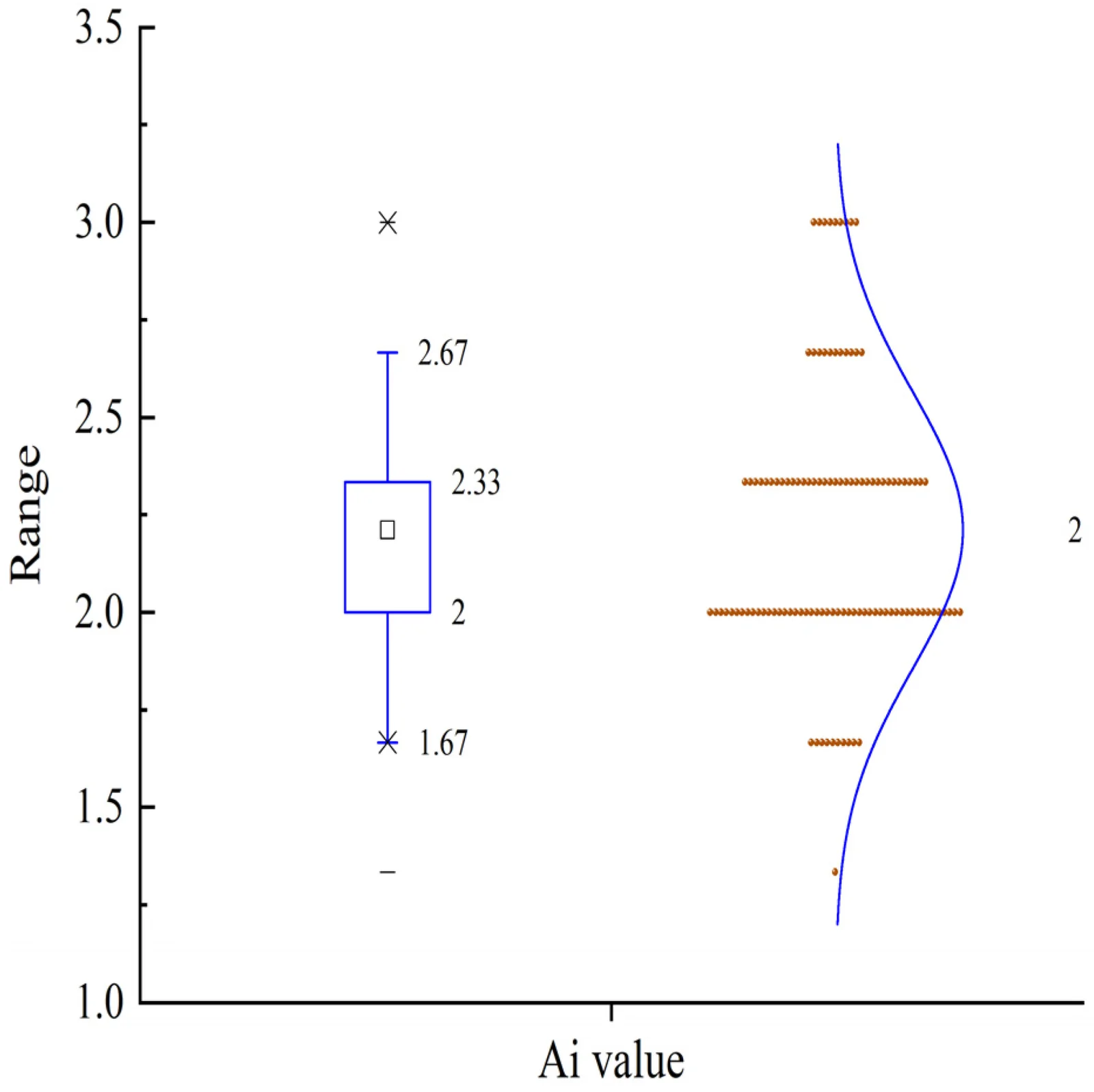
Distribution of *ai* values.

The data distribution (Figure 6) shows that 82.46% of the samples are highly concentrated in the intervals of *Ai* = 2 (42.10%) and its adjacent 2–3 range (40.35%), exhibiting a significant unimodal distribution, indicating that the *Ai* values of the vast majority of species stabilize at a moderately above-average level. The proportions at both extremes (*Ai* < 2 and *Ai* = 3) are each less than 10%, with very light and approximately symmetrical tails. This pattern of expansion in the middle and contraction at both ends reveals that this batch of samples does not contain a large number of highly specialized obligate anemophilous species (*Ai* = 3 is scarce), nor does it contain many taxa that are completely unsuitable for anemophilous pollination (*Ai* < 2 is rare). This result further suggests that the pollen of landscape trees is mostly facultatively anemophilous (possessing both entomophilous and anemophilous characteristics).

**Figure 7:**
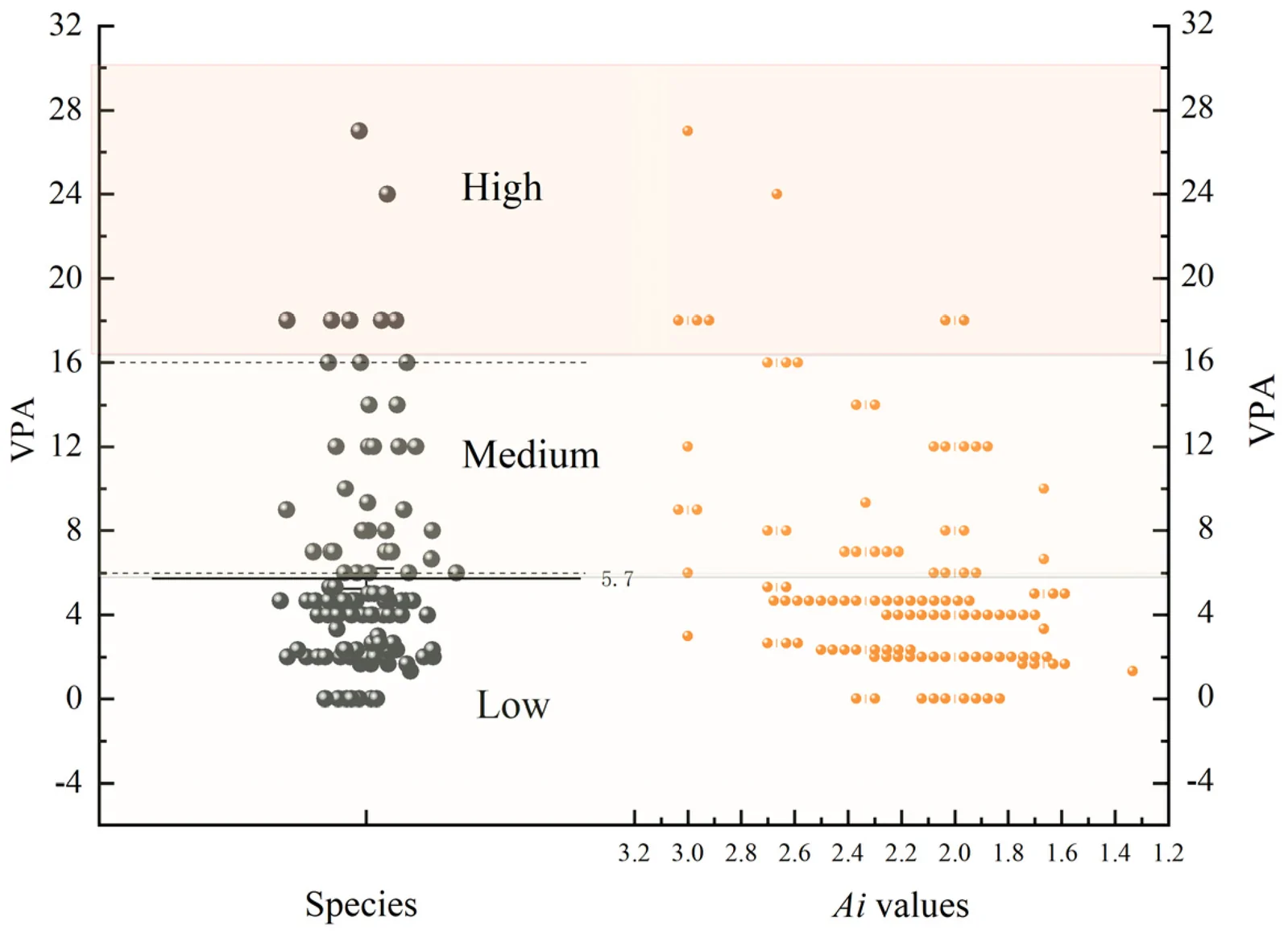
Distribution of *VPA* values and their corresponding *Ai* values.

The 114 tree species were classified based on their *VPA* values (Figure 7). Of these, 82 species were low-*VPA* species, accounting for 71.93%; 25 were medium-*VPA* species (21.93%); and 7 were high-*VPA* species (6.14%). Among the seven high-*VPA* species, gymnosperms (Pinaceae, Taxodiaceae, and Cupressaceae) predominated, accounting for five species (71.43%), while angiosperms (Fagaceae) comprised two species (28.57%). The *Ai* values of highly allergenic species fell within the range of 2–3. Specifically, values between 2.67 and 3 were observed in 71% of these species. In terms of flowering phenology, five of the seven high-*VPA* species (71.40%) initiated flowering in late winter, while the remaining two species flowered in late March. All seven species had a flowering duration exceeding 30 days. The overlapping flowering periods of these species resulted in a sustained peak of high-intensity pollen exposure from early to mid-spring.

The 25 medium-*VPA* species belonged to 21 families and 24 genera, of which angiosperms overwhelmingly predominated (21 species, 84%), whereas gymnosperms accounted for only 4 species (16%), belonging to Cupressaceae, Ginkgoaceae, and Taxodiaceae. The family and genus composition of medium-*VPA* species was highly dispersed, with no dominant family. The *Ai* values of medium-*VPA* species were predominantly concentrated within a narrow range of 2–2.67, accounting for approximately 80.77% of this category. Conversely, 59.26% of the low-*VPA* species showed *Ai* values not exceeding 2. The flowering period of medium-*VPA* species was mainly concentrated in spring (March to May, accounting for 72%). Among them, 15 species (60%) had a short flowering duration (≤30 days). However, *Ligustrum lucidum* had a flowering period starting in April and lasting for 80 days, spanning late spring to early summer. This flowering period overlapped in April with the late phase of the pollen season of high-*VPA* trees, thereby prolonging the composite pollen exposure peak from spring to early summer.

### 3.4. Spatial Distribution of Species Diversity and VPA Composition

In terms of species diversity (Table 3), the mean species richness across the six zones was 44.8, with a mean Shannon–Wiener index (*H′*) of 3.13 and a mean Pielou evenness (*J*) of 0.82, indicating a moderately high overall level of species diversity within the botanical garden. Across the entire garden, a total of 1196 low*-VPA*, 295 medium-*VPA*, and 91 high-*VPA* trees were recorded, accounting for 75.60%, 18.65%, and 5.75% of the total individual trees, respectively, demonstrating that low-*VPA* species dominate the landscape configuration (Table 4).

However, considerable spatial heterogeneity in diversity and *VP*A composition was observed among the six functional zones. High diversity with medium-VPA dominance was observed in Zones 2 and 4. The highest diversity was found in Zone 2 (*H′* = 3.95, *J* = 0.89), while values in Zone 4 were also above the garden-wide mean (*H′* = 3.25, *J* = 0.86). Their medium-*VPA* proportions ranked second highest (19.56%) and highest (24.09%) among all zones, respectively. Zone 1 and Zone 6 showed low diversity with low-*VPA* dominance, both showing the lowest diversity (*H′* = 2.63 and 2.68) and most uneven distributions (*J* = 0.71 and 0.70). Their low-*VPA* proportions reached 82.95% and 82.98%, respectively both exceeding the garden-wide mean (75.60%) and ranking highest among all zones. Zone 5 showed moderate diversity with high-*VPA* dominance, with diversity indices close to garden-wide means (*H′* = 3.28, *J* = 0.82), but its high-*VPA* proportion (10.81%, 55 species) was the highest across the garden, making it a hotspot for pollen allergenicity. Zone 3 had the lowest species richness (*R* = 23) and the lowest high-*VPA* proportion (1.96%, 1 species), making it the safest area for visitors with pollen allergies.

In summary, the botanical garden as a whole was dominated by low-*VPA* species (75.60%), indicating a relatively low overall allergenic potential at the species level. No simple linear relationship was found between species diversity (*R/H′/J*) and *VPA* composition. While this characterization provides a static background for understanding the potential allergenicity of each zone, *VPA* reflects the intrinsic allergenic capacity of species rather than actual atmospheric exposure levels. Consequently, one-year pollen concentration monitoring was subsequently conducted across all zones to verify the relationship between species-specific allergenic potential and actual airborne pollen exposure and to identify the seasons and areas requiring priority attention.

### 3.5. Temporal and Spatial Distribution of Pollen Concentration in the Botanical Garden

#### 3.5.1. Seasonal Variation Pattern of Pollen Concentration

For each of the 22 sampling sites, three replicate slides were exposed per sampling day, and the mean of the three slides represented one site–day data point. Over the 45-day sampling period, this yielded a theoretical total of 990 site–day data points (22 sites × 45 days), of which 979 valid site–day data points were obtained and used in the analysis. The remaining 11 site–day data points were excluded due to slide loss, slide damage, and sampling interruption caused by extreme weather.

**Figure 8:**
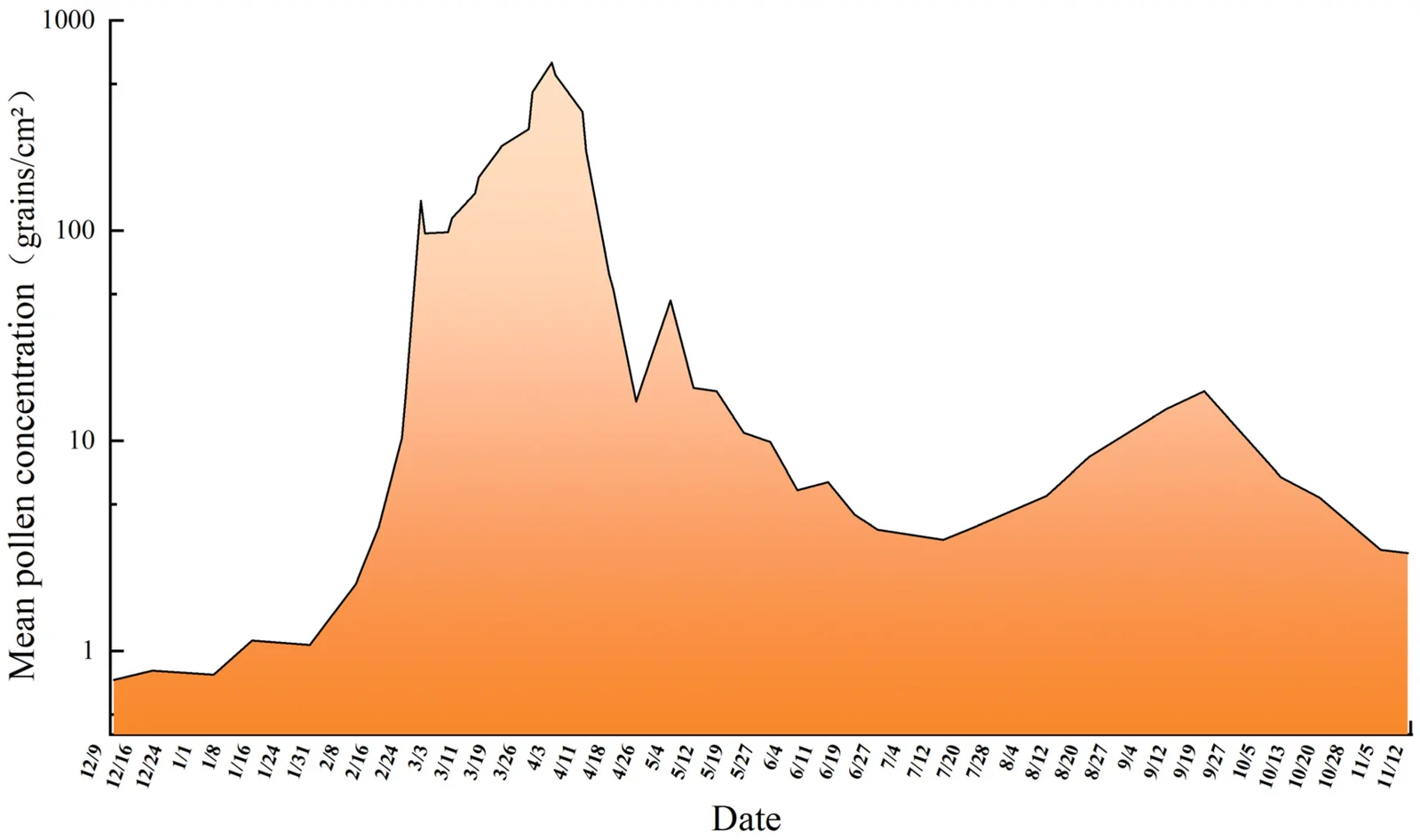
Daily mean pollen concentration on measurement days.

In spring (Figure 8), monthly averages (grains/cm^2^) ranked in the order: April (243.56) > March (207.47) > May (20.56); the peak occurred on April 3 (632.55) and the lowest value on May 30 (9.90). Except for May (Level 2), all spring months reached Level 5. In summer, monthly mean concentrations (grains/cm^2^) from high to low were: August (6.90) > June (5.01) > July (3.63). The highest concentration occurred on August 21, reaching 8.38 grains/cm^2^. In autumn, monthly concentration (grains/cm^2^) showed a decreasing trend from September (15.72) through October (6.06) to November (2.97), after a small peak in September. The peak occurred on September 20, reaching 17.30 grains/cm^2^. The September mean concentration falls into Level 2, indicating a relatively low allergy risk. In winter, from December to January, pollen levels were extremely low with no obvious trend. In early and mid-February, pollen levels began to increase slowly, followed by a sharp increase in late February, with the highest peak occurring on February 28 at 104.9 grains/cm^2^. The mean pollen concentration in February has reached Level 3, indicating a moderate allergy risk. Overall, from a temporal perspective, pollen concentration in the botanical garden exhibits a pronounced primary peak in spring and a much weaker secondary rise in autumn.

#### 3.5.2. Spatial Distribution of Pollen Concentration

**Figure 9:**
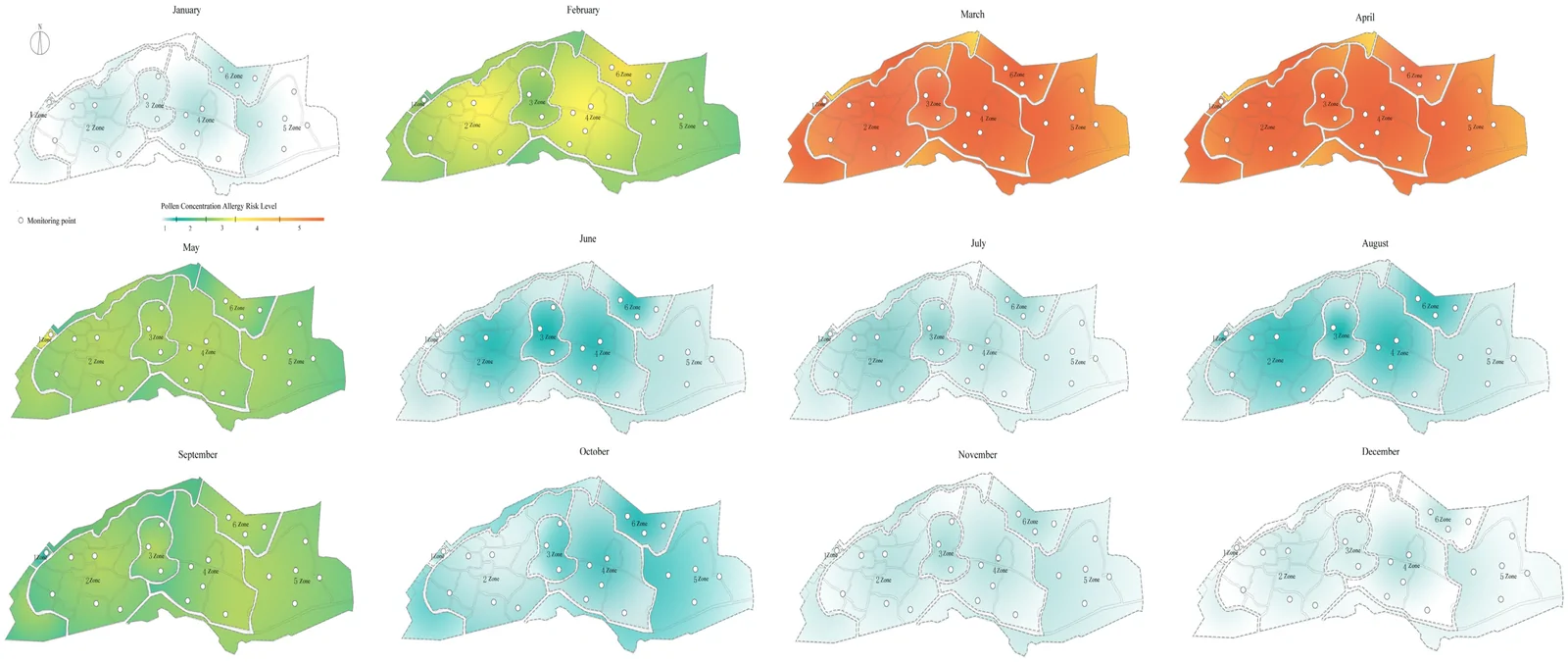
Variation of pollen concentration in different functional zones of the botanical garden.

Based on the seasonal pollen concentration data from each zone and the allergy risk classification, a spatiotemporal analysis was conducted (Figure 9). In spring (March and April), all zones recorded mean concentrations exceeding 80 grains/cm^2^, reaching Level 5 (extremely high risk). The highest concentrations were observed in Zone 2 (themed specialized garden area), Zone 4 (ecological recreation area), and Zone 5 (ecological conservation area), with April values of 298.80, 265.20, and 262.00 grains/cm^2^, respectively. By May, concentrations in all zones decreased to Levels 2–3: Zone 1 (entrance service area) remained at Level 3 (30.40 grains/cm^2^), while the other zones fell to Level 2. However, Kruskal–Wallis tests revealed no significant differences among the six zones in spring (*p*> 0.05).

In summer, all zones had concentrations below 10 grains/cm^2^, placing them uniformly at Level 1 (low risk). Despite the uniformly low absolute values, a significant difference was detected (*H* = 12.269, *p* = 0.031). Pairwise comparisons with Bonferroni correction (adjusted *α* = 0.0033) indicated that Zone 3 (educational leisure area) had significantly higher concentrations than Zone 5 (*p* = 0.001), and Zone 6 (scientific research nursery area) also exhibited significantly higher concentrations than Zone 5 (*p* = 0.003). No other pairwise differences were significant.

In autumn, only in September did Zones 2 to 6 reach Level 2 (relatively low risk), while all other zones remained at Level 1. The autumn differences among zones were not statistically significant (*p* > 0.05). In winter (February), a marked increase in pollen concentration (grains/cm^2^) was observed: Zone 2 (40.04), Zone 4 (40.48), and Zone 6 (42.77) reached Level 4 (high risk), whereas Zone 1 (18.60), Zone 3 (24.10), and Zone 5 (19.58) were at Level 2. No significant differences were found among zones in winter (*p* > 0.05). From a spatial perspective, Zones 2, 4, and 5 exhibited the highest overall annual allergy risk: Zones 2 and 4 reached Level 5 in spring and Level 4 in winter, while Zone 5 reached Level 5 in spring. Additionally, Zone 6 presented Level 4 risk in winter (February) and thus deserves attention.

#### 3.5.3. Spring Pollen Concentration and Meteorological Drivers

Since pollen concentrations in summer, autumn, and winter were generally below 40 grains/cm^2^ (Level 2 or 1), corresponding to low or relatively low risk levels, this study focused primarily on spring data (March–May, *n* = 21) and used Spearman correlation analysis and the generalized linear model (GLM) to explore the relationship between spring pollen concentration and meteorological factors. Spearman correlation analysis first revealed the association patterns between each meteorological factor and pollen concentration at the univariate level.

At the whole-garden scale, temperature variables (mean and maximum temperature) both showed significant negative correlations with pollen concentration (*r* = −0.470 and −0.527, respectively, *p* < 0.05), suggesting that temperature as a whole exerts a negative association with pollen concentration. Sunshine duration exhibited even stronger correlations (whole-garden *r* = −0.644, *p* < 0.01) and more consistent spatial patterns: it was highly significantly negatively correlated with all functional zones (especially Zones 2–6 and the whole garden), with correlation coefficients ranging from −0.428 to −0.725 (*p* < 0.01), suggesting that sunshine duration may be the most stable negative driving factor across the entire garden. In contrast, mean wind speed did not show significant correlations in any zone (correlation coefficients ranging from 0.021–0.103, all non-significant), and diurnal temperature range showed weak correlations only in individual zones (e.g., Zone 6).

The GLM, after controlling for the effects of other meteorological factors, further validated and refined the above findings. Because mean and maximum temperature were collinear with sunshine duration (*VIF* > 10), they were not included in the GLM. As shown in Table 5 and Table 6, sunshine duration remained a significant negative independent predictor in the multivariate model (*B* = −0.022, *p* < 0.001), with the largest Wald χ^2^ value (19.624). In contrast, mean wind speed showed a significant positive effect in the GLM (*B* = 3.604, *p* = 0.038), which does not contradict its non-significant result in the Spearman analysis. Spearman analysis reflects the net association between single variables, whereas the GLM, after controlling for confounding factors such as temperature and sunshine duration, revealed that wind speed has an independent promoting effect on pollen concentration (possibly through horizontal transport or surface resuspension). However, diurnal temperature range did not show a significant independent effect in the multivariate model (*B* = 0.03, *p* = 0.70), suggesting that the negative effects of temperature variables observed in the Spearman analysis were largely mediated by sunshine duration, rather than reflecting a direct causal influence of temperature variation.

## 4. Discussion

### 4.1. Allergenic Characteristics of Landscape Trees Revealed by an Improved VPA Index

Our results indicate that tree species selection in the Chengdu Botanical Garden is generally oriented toward low allergenicity. Based on species richness, Low-*VPA* species accounted for 75.60% of total landscape trees, while medium- and high-*VPA* species together comprised only 24.5%, among which high-*VPA* individual numbered only 91, representing 5.75% of all landscape tree species. This low-*VPA* profile stands in sharp contrast to that of most urban green spaces. For example, the proportion of tree species with medium- and high-*VPA* in Chengdu People’s Park reaches 64% (with high-*VPA* species accounting for 40%) [47]; in García Comprehensive Park (Spain), the proportion is 44.13% [30]; and in three urban parks in Bratislava, the proportion ranges from 36.9% to 50%, while the proportion of high-*VPA* species ranges from 22.6% to 30.6% [48]. For roadside green spaces, the proportion of high-*VPA* tree species in the old districts of Chengdu is 25.5% [23].

This difference stems from distinct planning objectives between botanical gardens and general urban green spaces. Botanical gardens are primarily designed for species diversity conservation, science popularization, and germplasm collection. Their introduction strategies emphasize taxonomic representativeness rather than landscape homogeneity. Consequently, a large number of families and genera that are less commonly used in routine urban greening have been introduced. Among these taxa, the proportion of entomophilous or low-pollen-producing species is relatively high, which naturally dilutes the relative proportion of highly allergenic species. On the other hand, ordinary urban parks and street green spaces are often constrained by practical factors such as nursery market supply, the demand for rapid landscaping effects, and engineering costs, which limit the range of available species and make it difficult to achieve a comparable level of allergy risk management.

### 4.2. Seasonal Pollen Distribution Patterns and Their Implications for Phenological Configuration

As described in Section 3.2, the flowering phenology of the botanical garden is highly concentrated, with 91.22% of species flowering from February to May. This extreme concentration has important implications for pollen exposure. From the perspectives of pollen allergy prevention and phenological diversity, such a compressed flowering schedule represents a marked structural imbalance. Instead of being staggered across seasons, the majority of floral resources, and consequently pollen emissions, are packed into a brief spring window, leaving other seasons with minimal flowering activity. This pattern lacks the temporal dilution effect that a more evenly distributed phenology would provide, and therefore offers limited ecological buffering against concentrated pollen loads.

Moreover, the early onset of this concentrated phenology further amplifies the risk. By mid- to late February, more than one-fifth of the landscape trees had already entered flowering, and the cumulative progression of early-spring species, as documented in Section 3.2, creates a sustained ramp-up in pollen emission well before the main spring peak. This early start is not a local peculiarity. Compared with People’s Park in the same city, the peak flowering of the earliest-flowering species in the botanical garden occurred approximately 10 days earlier [47], suggesting that the advanced pollen season is a distinctive feature of this botanical garden rather than a general urban green space phenomenon. This temporal advance extends the spring pollen exposure window and increases the cumulative allergenic load for sensitive visitors, particularly those who visit the garden during late winter and early spring.

Compared with other urban green spaces, the spring pollen concentration at the botanical garden was relatively high. Taking People’s Park [47], also in Chengdu, as an example, its mean daily pollen concentration in March was 45.54 grains/cm^2^, markedly lower than the botanical garden’s March mean (207.47 grains/cm^2^) and also below its spring mean (176.71 grains/cm^2^). In September, People’s Park’s mean daily concentration was 10.73 grains/cm^2^ [47], likewise lower than the botanical garden’s September mean (15.72 grains/cm^2^). Moreover, according to phenological observations at the two parks, the peak flowering period of the first-flowering species in the botanical garden occurred approximately 10 days earlier than that in People’s Park [47]. Because this phenological comparison is less sensitive to interannual variation in pollen concentration, it provides independent support for the earlier onset of the pollen season at the botanical garden. In addition, the mean spring pollen concentration in green spaces of Beijing has been reported to be below 47 grains/cm^2^ [49], markedly lower than the botanical garden’s spring mean. The maximum pollen concentration on the north shore of Kunming Lake in the Summer Palace during March–April was 79.79 grains/cm^2^ [50], and the corresponding maximum at Temple Heaven Park was 165.06 grains/cm^2^ [51], both lower than the botanical garden’s spring mean. Given the differing observation years and in some cases sampling protocols, these differences should be interpreted with caution.

The above results adds another dimension the conclusion drawn in Section 4.1 that the botanical garden has a low-*VPA* composition. Although high-*VPA* species accounted for only 6.14%, the spring pollen concentration was obviously higher than that of the same-city People’s Park and most green spaces in Beijing. This can be explained from two perspectives. First, although ordinary urban parks have a higher proportion of high-*VPA* species, the flowering periods of the selected trees are often staggered, as landscape design typically aims for a four-season visual effect. In contrast, 91.22% of the trees in the botanical garden bloom synchronously within a relatively short four-month window (especially from late February to May), and this overlapping effect leads to a sharp increase in local atmospheric pollen load over a short period, forming a pollen concentration peak. Second, the trees in the botanical garden are mostly mature, unpruned large specimens with much greater individual flower production than the frequently pruned street trees in urban roads. This indicates that a low-*VPA* species composition does not necessarily equate to low pollen exposure; phenological synchrony and individual pollen output play equally critical roles in determining the actual allergy risk. Therefore, in urban green space management, it is necessary to establish independent, category-specific early warning thresholds for specialized green spaces such as botanical gardens. Otherwise, regional background values may not reflect the true conditions of local pollen source areas.

### 4.3. Meteorological Controls on Spring Pollen

Unlike many studies that emphasize temperature as the dominant factor [52,53,54], our study found that although temperature variables exert a significant negative correlation on pollen concentration in the univariate analysis, sunshine duration is the most robust core factor negatively associated with spring pollen concentration, while mean wind speed acts as an independent promoting factor after controlling for other meteorological conditions. This result can be corroborated from a phenological perspective.

Sunshine duration, a proxy for photoperiod, serves as a core and stable signal driving the spring phenological onset of temperate woody plants [55]. Using phenological data from 102 sites across China’s monsoon region for four deciduous broadleaved tree species over the period 1981–2014, Lang et al. [56] demonstrated that photoperiod extension triggered 80.8% of leaf bud and 77.7% of flower bud growth initiation events, with this proportion increasing markedly from north to south, while the proportion induced by temperature showed a concurrent decreasing trend along the same climatic gradient. This indicates that in southern regions with short, mild winters, extended spring daylength can compensate for insufficient chilling accumulation to initiate bud growth. Chengdu, situated at the southern end of this gradient (low latitude), experiences a relatively small temperature difference between winter and spring, which further amplifies the role of photoperiod as a stable cue. Consequently, the influence of sunshine duration outweighs that of temperature in this region.

Notably, the significant negative correlations of both temperature and sunshine duration with pollen concentration do not indicate direct physiological inhibition. Instead, they arise from a temporal mismatch: the peak periods of spring temperature and sunshine duration in Chengdu (late April to May) occur after the main pollen release window (March–April) of most tree species, when the pollen source is already exhausted, leading to a statistical negative association. This pattern has been validated across different plant taxa, including Poaceae, *Quercus*, and *Pinus* in Poland, Spain [57], and Kunming, China [58]. Although a mechanistic explanation for the observed negative correlations is provided by this temporal mismatch, the contribution of phenological progression cannot be fully separated from meteorological effects.

The negative correlation with sunshine duration was significant in all six functional zones (*r* = −0.428 to −0.725, *p* < 0.01), indicating that this association was not driven by incidental fluctuations in a single pooled series, but represented a pattern that was consistently observed across the entire garden. However, because a stable negative correlation could also be generated by the flowering phenological progression within each zone, the contribution of phenological progression cannot be fully excluded based on this result alone.

The role of wind speed warrants further discussion. Spearman correlation analysis showed no significant correlation between mean wind speed and pollen concentration in any of the functional zones. However, after controlling for confounding factors such as temperature and sunshine duration in the generalized linear model (GLM), mean wind speed exhibited a significant positive effect. This discrepancy suggests that the true effect of wind speed is masked at the univariate level by strong seasonal trend signals. Specifically, the substantial seasonal variation in dominant factors such as sunshine duration and temperature accounts for the majority of variance in pollen concentration, while wind speed, a relatively stable variable, has its subtle transport effect that is difficult to detect in isolation. Only after these dominant seasonal signals are statistically controlled for in the GLM does the independent contribution of wind speed to pollen concentration variation become evident.

The above findings provide clear implications for the construction of pollen prediction models for urban green spaces. First, greater emphasis should be placed on photoperiod indicators such as sunshine duration, incorporating them as core signals of phenological onset into models, rather than focusing solely on more variable meteorological variables like temperature. Second, stage-specific modeling based on phenological phases should be adopted. The entire spring should be divided into the initiation, peak, and decline phases of pollen release, and separate prediction sub-models should be established for each phase. Particular attention should also be paid to separating phenological effects from meteorological effects. Phenological progression indicators, such as flowering onset, full bloom, and the end of flowering, should be explicitly incorporated, or approaches such as hierarchical models, structural equation models, and time-series decomposition should be employed to disentangle the effects of phenological progression from those of meteorological factors including temperature, sunshine duration, and wind speed. In this way, distortions in statistical relationships caused by pooling full-season data can be avoided, and the independent contribution of each factor can be more accurately assessed. Third, non-significant factors in univariate analysis should not be simply dismissed as irrelevant, and multivariate statistical approaches are essential for identifying independent effects in complex phenological–meteorological relationships.

### 4.4. Tree Species Management Strategies Based on the Exposure Concentration and Tree VPA

Although pollen concentration monitoring has revealed spatiotemporal heterogeneity in exposure levels, which—as noted earlier—reflect airborne pollen exposure intensity rather than clinically validated allergy risk, concentration data alone remain insufficient to guide precise interventions, because under identical total concentrations, the proportion of high-*VPA* versus low-*VPA* species exerts substantially different health threats to sensitive populations. It is therefore necessary to integrate the pollen concentrations measured across functional zones with the VPA class composition of tree species.

Based on this framework, the six functional zones can be classified into five heterogeneous risk types. The highest proportion of high *VPA* individual trees (10.81%) is found in Zone 5 (ecological conservation area), with spring concentrations reaching Level 5, qualifying it as a high-risk core zone, where the enrichment of highly allergenic gymnosperms is the fundamental source of risk. The highest spring peak concentrations across the garden (298.80 and 265.20 grains/cm^2^, respectively) are recorded in Zones 2 and 4, coupled with moderately elevated proportions of medium-*VPA* individual trees (19.56% and 24.09%). The cumulative effect of these two factors has driven up local pollen allergy risk, designating them as concentration-dominated secondary risk zones. Zone 6, despite having 82.98% low-VPA individual trees and thus being evaluated as the safest area based on species inventory, exhibits a measured winter (February) concentration of 42.77 grains/cm^2^, reaching Level 4 and tying with Zones 2 and 4 for the highest winter levels. A marked mismatch between *VPA* composition and measured concentration is observed in Zone 6, placing it as an individually driven medium- to high-risk zone. This discrepancy also exposes a fundamental limitation of *VPA* assessment, which is based on species morphological traits and completely overlooks inter-individual variation in pollen production. Zone 1 has 82.95% low-*VPA* species, with moderate concentration levels in both winter and spring and short visitor residence times, qualifying it as a low-exposure risk zone. Zone 3, although also reaching Level 5 in spring due to garden-wide background pollen dispersion with no significant difference from other zones, maintains low risk levels in all other seasons. Moreover, its high-*VPA* individual trees account for only 1.96%, the lowest proportion in the garden. Therefore, considering the entire annual cycle, Zone 3 is a relatively suitable recreational area for allergy-sensitive visitors.

Furthermore, the diversity indices provide another important caveat. The highest species richness (*R* = 87) and the highest Shannon–Wiener diversity (*H′*= 3.95) were found in Zone 2, and the highest spring peak pollen concentration was also recorded there. The positive association between these two variables stems from the pronounced phenological concentration, with 91.22% of all species in the garden flower from February to May, meaning that zones with larger species pools harbor a greater absolute number of simultaneously flowering taxa. This indicates that under conditions of high phenological overlap, increased diversity generates an accumulation effect rather than a dilution effect in terms of pollen source intensity. Therefore, species diversity per se cannot be used as a direct proxy for allergenic risk: the critical determinants are the *VPA* class of dominant species and their per-individual pollen output.

Based on the above risk classification, differentiated management strategies should be formulated for each functional zone in accordance with their seasonal risk profiles. Unlike ordinary urban green spaces, botanical gardens are mandated with species conservation and science popularization, and are characterized by a high proportion of native and rare or endangered tree species. Consequently, the core objective of management is not to eliminate allergenic species altogether, but rather to strike a balance between ecological conservation and public health through a combination of physical barriers, phenological regulation, and visitor flow guidance.

In spring, all zones should activate the highest level of pollen allergy alert, disseminating concentration data and protective recommendations in real time via on-screen displays and official social media accounts. On the basis of this unified warning system, zone-specific measures should be implemented according to their respective risk characteristics. In Zone 5, the high-risk core zone, it is recommended to establish low, dense, evergreen hedgerows between the conservation area and the main visitor routes; these hedgerows can modify near-surface airflow patterns and promote local pollen deposition, thereby partially mitigating downwind dispersal toward recreational areas. In parallel, pre-flowering pruning and water spraying to increase humidity should be applied to high-*VPA* species. Zones 2 and 4 have the highest spring peak concentrations, attributable to their large species pools and highly synchronized flowering phenology. The dominant species that contribute most to the risk should be relocated to other zones or to low-exposure areas such as Zone 1. This recommendation is justified because these two zones primarily serve exhibition and recreation functions, rather than irreplaceable germplasm conservation. For individuals not scheduled for relocation, pre-flowering pruning can be implemented. Zone 6 requires a comprehensive inventory of large-aged gymnosperm individuals and pre-flowering pruning, coupled with localized spray dust suppression in areas where research personnel are concentrated during high-concentration periods. Zone 3, which has the lowest background allergenic potential, can be promoted as a priority recreational route for sensitive visitors during peak spring concentration periods. Zone 1 should continue with routine monitoring and public alert issuance.

## 5. Conclusions

Our findings indicate that whether assessed by species proportion or individual abundance, low-*VPA* species were found to constitute a relatively high proportion in the Chengdu Botanical Garden, and its allergenicity level was shown to be much lower than that of other parks in the same city and of European urban parks.

However, spring pollen concentrations in the garden were higher than reported urban park, and reached an extremely high risk level (Level 5). An early peak appeared in late February (Level 3). This was attributed to the highly concentrated flowering phenology. Specifically, 91.22% of all species flower from February to May, with 50.87% blooming in March and April. This creates a synchronous burst of pollen release. Species diversity analysis revealed that the mean *R*, *H*’, and *J* were 44.8, 3.13, and 0.82, respectively, indicating moderately high diversity. However, under conditions of high phenological overlap, increased diversity produced an accumulation effect rather than a dilution effect on pollen concentration. In addition, meteorological factors exerted significant influences on pollen concentration dynamics. Both temperature and sunshine duration showed significant negative correlations in univariate analyses. Sunshine duration was the most crucial factor affecting spring pollen concentration. Mean wind speed acted as an independent promoting factor after controlling for other meteorological conditions.

These findings reveal a key limitation: relying solely on species *VPA* value or solely on pollen concentration monitoring without integrating phenological and meteorological information may either misrepresent or fail to explain the true allergy risk in botanical gardens. Therefore, a risk framework that couples pollen concentration with *VPA* classes was established. Based on this framework, we categorized the six functional zones into five risk types. The ecological conservation area was identified as a high-risk core zone. The themed specialized garden area and the ecological recreation area were classified as concentration-dominated secondary risk zones. The entrance service area was designated as a low-exposure risk zone. The scientific research nursery area was identified as an individually driven medium- to high-risk zone, where a marked mismatch between *VPA* assessment and measured concentrations was observed. The educational leisure area was recognized as a relatively safe zone suitable for sensitive visitors throughout the year. Given the botanical garden’s dual mission of species conservation and public health, we recommend that management should not aim to eliminate allergenic species. Instead, it should achieve a balance between ecological conservation and health protection through integrated measures. These include visitor flow guidance, physical barriers, and phenological regulation.

In summary, this study confirms that the Chengdu Botanical Garden poses a substantial spring pollen allergy risk. However, this risk is concentrated from February to May and is driven primarily by phenological overlap rather than by high-*VPA* species. Effective risk management requires a shift from static species-based inventories to dynamic, multi-dimensional approaches. These approaches should integrate phenology, meteorology, real-time monitoring, and spatially differentiated interventions. Such management would allow botanical gardens to fulfill their conservation mandate while better safeguarding public health. We recommend extending this framework to other botanical gardens and urban green spaces to improve the accuracy of pollen allergy risk assessments across various green space types.

## Figures and Tables

**Table 1:** Scoring criteria for the anemophily index (*Ai*) based on pollen morphological traits.

Trait	Score = 3	Score = 2	Score = 1
Size index (*SI*)	20 μm ≤ *SI* ≤ 50 μm	10 μm ≤ *SI* < 20 μm or 51 μm < *SI* ≤ 70 μm	*SI* < 10 μm or *SI* > 70 μm
Shape index (Polar axis/equatorial axis, *P/E*)	0.88 < *P/E* < 1.14	0.50 ≤ *P/E* ≤ 0.88 or 1.14 ≤ *P/E* ≤ 2	*P/E* < 0.50 or *P/E* > 2
Exine ornamentation	psilate, granular, or verrucate–rugulate	striate, foveolate, or cerebroid	reticulate, echinate, tuberculate, or verrucate

**Table 2:** Classification of total pollen concentration levels and corresponding exposure intensity grades.

Allergy Risk Level	Pollen Concentration (Grain/cm^2^)	Description
1	≤10	low
2	10.1–25	relatively low
3	25.1–40	moderate
4	40.1–80	high
5	>80	extremely high

**Table 3:** Diversity of landscape trees in each function zone.

Function Zone	1	2	3	4	5	6	Mean
Richness index (*R*)	41	87	23	43	56	19	44.8
Shannon–Wiener index (*H’*)	2.63	3.95	2.98	3.25	3.28	2.68	3.13
Pielou index (*J*)	0.71	0.89	0.95	0.86	0.82	0.70	0.82

**Table 4:** Number and percentage of individual landscape trees in each VPA level by zone.

Zone	1	2	3	4	5	6
*VPA*	Number, Percentage	Number, Percentage	Number, Percentage	Number, Percentage	Number, Percentage	Number, Percentage
Low	180, 82.95	351, 77.14	41, 80.39	222, 73.27	363, 71.32	39, 82.98
Medium	26, 11.98	89, 19.56	9, 17.65	73,24.09	91, 17.88	7, 14.89
High	11, 5.07	15, 3.30	1, 1.96	8, 2.64	55, 10.81	1, 2.13

**Table 5:** Spearman rank correlation analysis between pollen concentration and meteorological factors in different zones.

Factors	Pollen Concentration in Different Zones						
	1	2	3	4	5	6	Entire Garden
Mean temperature	−0.174	−0.419	−0.394	−0.416	−0.518 *	−0.584 **	−0.470 *
Maximum temperature	−0.200	−0.477 *	−0.452 *	−0.479 *	−0.553 **	−0.647 **	−0.527 *
Minimum temperature	−0.090	−0.305	−0.303	−0.297	−0.490 *	−0.468 *	−0.371
Diurnal temperature range	−0.044	−0.283	−0.262	−0.305	−0.248	−0.453 *	−0.318
Mean relative humidity	0.168	−0.005	−0.042	0.105	0.052	0.212	0.099
Mean wind speed	0.095	0.086	0.103	0.073	0.021	0.082	0.105
Maximum wind speed	0.087	0.068	0.076	−0.016	−0.027	−0.104	0.001
Mean light intensity	−0.272	−0.364	−0.343	−0.362	−0.342	−0.460 *	−0.391
Sunshine duration	−0.428	−0.604 **	−0.612 **	−0.631 **	−0.683 **	−0.725 **	−0.644 **

Note: * *p* < 0.05; ** *p* < 0.01.

**Table 6:** Parameter estimates of the log-linear model for spring pollen concentration.

Factors	*B*	*SE*	95% CI for B	Hypothesis Testing	
				Wald *χ*^2^	*p*-Value
Diurnal temperature range	0.030	0.0789	−0.124, 0.185	0.149	0.700
Mean wind speed	3.604	1.7351	0.203, 7.005	4.315	0.038
Sunshine duration	−0.022	0.0051	−0.032, −0.013	19.624	0.000

Note: *B* = unstandardized coefficient; *SE* = standard error; CI = confidence interval. Dependent variable: ln (pollen + 1). GLM with normal distribution and identity link. Scale parameter = 0.778.

## Data Availability

The original data presented in the study are openly available on FigShare.

## Supplementary Materials

The following supporting information can be downloaded at https://www.mdpi.com/article/10.3390/plants15193063/s1, Table S1: Pollen morphological characteristics of the 114 landscape tree species; Table S2: Parameter assignments for *VPA* calculation.

## Abbreviations

The following abbreviations are used in this manuscript.

VPA	value of potential allergenicity
SI	size index
P/E	shape index (polar axis/equatorial axis)
Ai	anemophily index
dpp	duration of pollination period
ap	allergenic potential
FFD	first flowering date
EFD	end of flowering date
GLM	generalized linear model

## Appendix A

**Table A1:** Pollen morphological characteristics of the 114 landscape tree species.

0	Species	*SI* (Size) um	*P/E* (Shape)	Exine Ornamentation
1	*Ginkgo biloba*	22.86588721	2.104991372	striate
2	*Pinus thunbergii*	38.72286448	1.039575239	tuberculate
3	*Pinus massoniana*	45.4681199	1.068113439	tuberculate
4	*Cryptomeria japonica* var. *sinensis*	25.17539161	1.180815344	granular
5	*Taxodium mucronatum*	21.06417922	1.072331557	tuberculate
6	*Taxodium distichum* var. *imbricatum*	27.92764983	1.184208476	tuberculate
7	*Chamaecyparis pisifera*	27.91578967	1.004171184	granular
8	*Juniperus chinensis*‘*Kaizuca*’	19.71264145	1.004514856	granular
9	*Juniperus formosana*	29.4413756	1.002325961	granular
10	*Yulania* × *denudata*	34.96952842	1.457087068	foveolate
11	*Yulania soulangeana*	40.83534223	1.645580981	foveolate
12	*Magnolia grandiflora*	51.3824501	1.581563126	foveolate
13	*Magnolia delavayi*	60.00859015	2.347183551	foveolate
14	*Magnolia zenii*	52.84335761	2.001193503	foveolate
15	*Manglietia grandis*	45.75772617	2.35170955	foveolate
16	*Michelia maudiae*	29.98743552	2.220590135	foveolate
17	*Liriodendron chinense*	45.755237	2.022714434	reticulate
18	*Cinnamomum japonicum*	33.07273752	1.132050129	echinate
19	*Cinnamomum camphora*	22.89079554	1.011528897	echinate
20	*Phoebe zhennan*	28.91848761	1.001349528	echinate
21	*Machilus nanmu*	28.89062472	1.007051206	echinate
22	*Lindera megaphylla*	29.63629007	1.003715838	echinate
23	*Lindera communis*	27.33153917	1.002410062	echinate
24	*Platanus acerifolia*	16.56053687	1.297112003	reticulate
25	*Grevillea robusta*	51.08751283	0.940666899	verrucate
26	*Liquidambar formosana*	33.7080557	1.102985149	reticulate
27	*Celtis sinensis*	31.19641633	0.882375613	granular
28	*Morus alba*	20.41734157	1.005786252	granular
29	*Morus cathayana*	17.38705529	1.459798053	granular
30	*Broussonetia papyrifera*	15.20105673	1.006202901	granular
31	*Pterocarya stenoptera*	27.72944481	0.898840294	granular
32	*Castanea mollissima*	13.41653498	1.834335405	foveolate
33	*Castanopsis fissa*	14.2733099	1.752321637	foveolate
34	*Quercus acutissima*	28.10233067	1.379106472	tuberculate
35	*Quercus variabilis*	35.04042004	1.460685859	tuberculate
36	*Cyclobalanopsis glauca*	25.12006558	1.372106681	verrucate
37	*Alnus cremastogyne*	23.49908609	0.869093332	granular
38	*Casuarina equisetifolia*	31.27074229	0.835431791	striate
39	*Camellia synaptica*	47.44985825	1.586749851	foveolate
40	*Firmiana simplex*	31.90789006	1.276002378	reticulate
41	*Brachychiton acerifolius*	31.86118974	1.538224587	reticulate
42	*Reevesia pubescens*	26.42211467	1.140887862	reticulate
43	*Salix babylonica*	21.26001918	1.746106519	reticulate
44	*Diospyros lotus*	32.2586247	1.900679168	rugulate
45	*Styrax japonicus*	40.40838938	1.885286479	reticulate
46	*Sinojackia xylocarpa*	27.74908611	1.424233572	reticulate
47	*Melliodendron xylocarpum*	36.39588834	1.872261787	reticulate
48	*Chaenomeles cathayensis*	32.61652387	1.57997273	foveolate
49	*Photinia serratifolia*	26.63169874	1.868182937	striate
50	*Malus halliana*	33.71511845	1.981957988	striate
51	*Pyrus ussuriensis*	31.58274104	2.083796842	striate
52	*Prunus mume* f. *albo*-*plena‘Zi Di Bai’*	49.34497214	1.213034135	striate
53	*Prunus mume* f. *viridicalyx‘Bian lv e’*	48.26062185	1.151170179	striate
54	*Prunus mume* var. *bungo*	33.8345673	2.029718693	striate
55	*Prunus* × *blireana*‘*Meiren*’	32.53884954	2.099074346	striate
56	*Prunus campanulata*	39.55015332	1.892064817	striate
57	*Prunus* × *subhirtella*	36.73666082	1.25664948	striate
58	*Prunus campanulata*‘*Plena*’	39.27634495	1.482779561	striate
59	*Prunus*‘*Canton*’	34.22173757	1.153059112	striate
60	*Prunus serrulata*	30.87671475	1.664978685	striate
61	*Prunus* × *yedoensis*	34.65792868	1.767008824	striate
62	*Prunus cerasifera*	33.96606792	1.838771119	striate
63	*Prunus pseudocerasus*	34.58936531	1.82457113	striate
64	*Prunus cerasoides*	41.26717793	1.659305364	striate
65	*Prunus persica*‘*Duplex*’	47.56575443	2.002295174	striate
66	*Prunus persica*	42.05936417	1.258909708	striate
67	*Acacia podalyriifolia*	41.25470286	1.200925026	rugulate
68	*Acacia mearnsii*	38.91239824	1.000580866	rugulate
69	*Albizia kalkora*	76.10902371	1.276018985	rugulate
70	*Zenia insignis*	30.99237972	1.375317758	reticulate
71	*Bauhinia variegata*	45.10209704	1.555592358	striate
72	*Robinia pseudoacacia*	27.12279971	1.419297598	foveolate
73	*Erythrina variegata*	37.66379302	0.851158008	reticulate
74	*Erythrina corallodendron*	29.62213132	0.828453343	reticulate
75	*Punica granatum*	25.32272949	1.317057268	rugulate
76	*Lagerstroemia indica*	36.7410588	1.343138738	tuberculate
77	*Syzygium jambos*	18.24477649	0.638270048	verrucate
78	*Syzygium nervosum*	21.65090421	0.63680162	tuberculate
79	*Callistemon viminalis*	15.73991039	0.637277868	verrucate
80	*Melaleuca cajuputi* subsp. *cumingiana*	11.53814197	0.604964721	rugulate
81	*Acca sellowiana*	18.12956172	0.609590692	verrucate
82	*Davidia involucrata*	31.31821569	1.716330965	foveolate
83	*Alangium chinense*	52.85489618	0.546016242	tuberculate
84	*Cornus wilsoniana*	35.20156782	1.461869931	rugulate
85	*Cornus capitata*	26.78002606	1.470146213	granular
86	*Cornus controversa*	40.94622994	1.76098259	tuberculate
87	*Euonymus maackii*	32.78547436	1.401025256	reticulate
88	*Mallotus tenuifolius*	22.04110228	1.163116548	echinate
89	*Triadica sebifera*	36.14095086	1.684644533	foveolate
90	*Bischofia javanica*	22.57224948	1.579084415	reticulate
91	*Koelreuteria paniculata*	25.09939875	1.753205702	striate
92	*Sapindus saponaria*	19.8313534	1.86658586	foveolate
93	*Aesculus chinensis* var. *wilsonii*	25.45222955	1.666893235	striate
94	*Acer catalpifolium*	30.16853765	1.750026272	striate
95	*Acer palmatum*	28.89252212	1.880873964	striate
96	*Acer buergerianum*	30.29705623	1.840608383	striate
97	*Toxicodendron vernicifluum*	24.70232786	1.330322964	striate
98	*Rhus potaninii*	30.64105703	2.128939501	striate
99	*Choerospondias axillaris* var. *pubinervis*	29.3605487	1.528955385	striate
100	*Melia azedarach*	50.41376159	1.050493762	foveolate
101	*Chukrasia tabularis*	28.22171602	1.431386491	foveolate
102	*Toona sinensis*	23.22581163	1.141610818	psilate
103	*Brassaiopsis glomerulata*	31.43422371	1.221716689	reticulate
104	*Brugmansia arborea*	45.2288526	0.884435002	striate
105	*Vitex negundo* var. *cannabifolia*	26.42339574	1.683103634	foveolate
106	*Fraxinus rhynchophylla*	20.98691595	1.157479429	reticulate
107	*Fontanesia phillyreoides subsp*. *fortunei*	18.3420466	1.365348136	reticulate
108	*Ligustrum lucidum*	27.85882869	1.529286995	reticulate
109	*Paulownia fortunei*	22.34455493	1.914267324	reticulate
110	*Jacaranda mimosifolia*	50.55247779	1.975093379	psilate
111	*Trachycarpus fortunei*	18.68380391	1.87919109	reticulate
112	*Livistona chinensis*	20.43906504	1.89070149	tuberculate
113	*Ehretia macrophylla*	29.87947476	1.931426711	tuberculate
114	*Crateva formosensis*	22.66983895	1.861491281	foveolate
