# Supplementary material for: Do Botanical Gardens Pose a Pollen Allergenicity Risk? A Multi-Method Assessment Integrating Species-Specific Values of Potential Allergenicity and Pollen Concentration Monitoring
Source: Plants (Basel). 2026 Oct 7;15(19):3063. doi: 10.3390/plants15193063 (PMC13667128; doi:10.3390/plants15193063)
Supplement: Supplementary file 1 [file plants-15-03063-s001.zip › plants-4546281-supplementary.pdf]

Table S1 Pollen morphological characteristics of the 114 landscape tree species

Num	Species	Size index（*SI*, um）	Polar axis / Equatorial axis (*P/E*)	Exine ornamentation
1	*Ginkgo biloba*	22.866	2.105	striate
2	*Pinus thunbergii*	38.723	1.040	tuberculate
3	*Pinus massoniana*	45.468	1.068	tuberculate
4	*Cryptomeria japonica* var. *sinensis*	25.175	1.181	granular
5	*Taxodium mucronatum*	21.064	1.072	tuberculate
6	*Taxodium distichum* var. *imbricatum*	27.928	1.184	tuberculate
7	*Chamaecyparis pisifera*	27.916	1.004	granular
8	*Juniperus chinensis*'*Kaizuca*'	19.713	1.005	granular
9	*Juniperus formosana*	29.441	1.002	granular
10	*Yulania* × *denudata*	34.970	1.457	foveolate
11	*Yulania soulangeana*	40.835	1.646	foveolate
12	*Magnolia grandiflora*	51.382	1.582	foveolate
13	*Magnolia delavayi*	60.009	2.347	foveolate
14	*Magnolia zenii*	52.843	2.001	foveolate
15	*Manglietia grandis*	45.758	2.352	foveolate
16	*Michelia maudiae*	29.987	2.221	foveolate
17	*Liriodendron chinense*	45.755	2.023	reticulate
18	*Cinnamomum japonicum*	33.073	1.132	echinate
19	*Cinnamomum camphora*	22.891	1.012	echinate
20	*Phoebe zhennan*	28.918	1.001	echinate
21	*Machilus nanmu*	28.891	1.007	echinate
22	*Lindera megaphylla*	29.636	1.004	echinate
23	*Lindera communis*	27.332	1.002	echinate
24	*Platanus acerifolia*	16.561	1.297	reticulate
25	*Grevillea robusta*	51.088	0.941	verrucate
26	*Liquidambar formosana*	33.708	1.103	reticulate
27	*Celtis sinensis*	31.196	0.882	granular

28	*Morus alba*	20.417	1.006	granular
29	*Morus cathayana*	17.387	1.460	granular
30	*Broussonetia papyrifera*	15.201	1.006	granular
31	*Pterocarya stenoptera*	27.729	0.899	granular
32	*Castanea mollissima*	13.417	1.834	foveolate
33	*Castanopsis fissa*	14.273	1.752	foveolate
34	*Quercus acutissima*	28.102	1.379	tuberculate
35	*Quercus variabilis*	35.040	1.461	tuberculate
36	*Cyclobalanopsis glauca*	25.120	1.372	verrucate
37	*Alnus cremastogyne*	23.499	0.869	granular
38	*Casuarina equisetifolia*	31.271	0.835	striate
39	*Camellia synaptica*	47.450	1.587	foveolate
40	*Firmiana simplex*	31.908	1.276	reticulate
41	*Brachychiton acerifolius*	31.861	1.538	reticulate
42	*Reevesia pubescens*	26.422	1.141	reticulate
43	*Salix babylonica*	21.260	1.746	reticulate
44	*Diospyros lotus*	32.259	1.901	rugulate
45	*Styrax japonicus*	40.408	1.885	reticulate
46	*Sinojackia xylocarpa*	27.749	1.424	reticulate
47	*Melliodendron xylocarpum*	36.396	1.872	reticulate
48	*Chaenomeles cathayensis*	32.617	1.580	foveolate
49	*Photinia serratifolia*	26.632	1.868	striate
50	*Malus halliana*	33.715	1.982	striate
51	*Pyrus ussuriensis*	31.583	2.084	striate
52	*Prunus mume* f. *albo-plena'Zi Di Bai'*	49.345	1.213	striate
53	*Prunus mume* f. *viridicalyx'Bian lv e'*	48.261	1.151	striate
54	*Prunus mume* var. *bungo*	33.835	2.030	striate
55	*Prunus × blireana'Meiren'*	32.539	2.099	striate
56	*Prunus campanulata*	39.550	1.892	striate

57	*Prunus* × *subhirtella*	36.737	1.257	striate
58	*Prunus campanulata*‘*Plena*’	39.276	1.483	striate
59	*Prunus*‘*Canton*’	34.222	1.153	striate
60	*Prunus serrulata*	30.877	1.665	striate
61	*Prunus* × *yedoensis*	34.658	1.767	striate
62	*Prunus cerasifera*	33.966	1.839	striate
63	*Prunus pseudocerasus*	34.589	1.825	striate
64	*Prunus cerasoides*	41.267	1.659	striate
65	*Prunus persica*‘*Duplex*’	47.566	2.002	striate
66	*Prunus persica*	42.059	1.259	striate
67	*Acacia podalyriifolia*	41.255	1.201	rugulate
68	*Acacia mearnsii*	38.912	1.001	rugulate
69	*Albizia kalkora*	76.109	1.276	rugulate
70	*Zenia insignis*	30.992	1.375	reticulate
71	*Bauhinia variegata*	45.102	1.556	striate
72	*Robinia pseudoacacia*	27.123	1.419	foveolate
73	*Erythrina variegata*	37.664	0.851	reticulate
74	*Erythrina corallodendron*	29.622	0.828	reticulate
75	*Punica granatum*	25.323	1.317	rugulate
76	*Lagerstroemia indica*	36.741	1.343	tuberculate
77	*Syzygium jambos*	18.245	0.638	verrucate
78	*Syzygium nervosum*	21.651	0.637	tuberculate
79	*Callistemon viminalis*	15.740	0.637	verrucate
80	*Melaleuca cajuputi* subsp. *cumingiana*	11.538	0.605	rugulate
81	*Acca sellowiana*	18.130	0.610	verrucate
82	*Davidia involucrata*	31.318	1.716	foveolate
83	*Alangium chinense*	52.855	0.546	tuberculate
84	*Cornus wilsoniana*	35.202	1.462	rugulate
85	*Cornus capitata*	26.780	1.470	granular

86	*Cornus controversa*	40.946	1.761	tuberculate
87	*Euonymus maackii*	32.785	1.401	reticulate
88	*Mallotus tenuifolius*	22.041	1.163	echinate
89	*Triadica sebifera*	36.141	1.685	foveolate
90	*Bischofia javanica*	22.572	1.579	reticulate
91	*Koelreuteria paniculata*	25.099	1.753	striate
92	*Sapindus saponaria*	19.831	1.867	foveolate
93	*Aesculus chinensis* var. *wilsonii*	25.452	1.667	striate
94	*Acer catalpifolium*	30.169	1.750	striate
95	*Acer palmatum*	28.893	1.881	striate
96	*Acer buergerianum*	30.297	1.841	striate
97	*Toxicodendron vernicifluum*	24.702	1.330	striate
98	*Rhus potaninii*	30.641	2.129	striate
99	*Choerospondias axillaris* var. *pubinervis*	29.361	1.529	striate
100	*Melia azedarach*	50.414	1.050	foveolate
101	*Chukrasia tabularis*	28.222	1.431	foveolate
102	*Toona sinensis*	23.226	1.142	psilate
103	*Brassaiopsis glomerulata*	31.434	1.222	reticulate
104	*Brugmansia arborea*	45.229	0.884	striate
105	*Vitex negundo* var. *cannabifolia*	26.423	1.683	foveolate
106	*Fraxinus rhynchophylla*	20.987	1.157	reticulate
107	*Fontanesia phillyreoides subsp. fortunei*	18.342	1.365	reticulate
108	*Ligustrum lucidum*	27.859	1.529	reticulate
109	*Paulownia fortunei*	22.345	1.914	reticulate
110	*Jacaranda mimosifolia*	50.552	1.975	psilate
111	*Trachycarpus fortunei*	18.684	1.879	reticulate
112	*Livistona chinensis*	20.439	1.891	tuberculate
113	*Ehretia macrophylla*	29.879	1.931	tuberculate
114	*Crateva formosensis*	22.670	1.861	foveolate

Table S2 Parameter assignments for VPA calculation

Num	Species	Duration of pollination period (dpp)	Allergenic potential (ap)	*SI*	*P/E*	Exine ornamentation	*Ai*	VPA
1	*Ginkgo biloba*	2	3	3	1	2	2	12
2	*Pinus thunbergii*	2	3	3	3	3	3	18
3	*Pinus massoniana*	2	3	3	3	3	3	18
4	*Cryptomeria japonica* var. *sinensis*	3	3	3	2	3	2.67	24
5	*Taxodium mucronatum*	3	3	3	3	3	3	27
6	*Taxodium distichum* var. *imbricatum*	2	3	3	2	3	2.67	16
7	*Chamaecyparis pisifera*	1	3	3	3	3	3	9
8	*Juniperus chinensis*'*Kaizuca*'	2	3	2	3	3	2.67	16
9	*Juniperus formosana*	2	3	3	3	3	3	18
10	*Yulania* × *denudata*	2	1	3	2	1	2	4
11	*Yulania soulangeana*	2	1	3	2	1	2	4
12	*Magnolia grandiflora*	2	1	2	2	2	2	4
13	*Magnolia delavayi*	1	1	2	1	2	1.67	1.67
14	*Magnolia zenii*	1	1	2	1	1	1.33	1.33
15	*Manglietia grandis*	1	1	3	1	1	1.67	1.67
16	*Michelia maudiae*	3	1	3	1	2	2	6
17	*Liriodendron chinense*	1	1	3	1	1	1.67	1.67
18	*Cinnamomum japonicum*	1	2	3	3	1	2.33	4.67
19	*Cinnamomum camphora*	1	2	3	3	1	2.33	4.67
20	*Phoebe zhennan*	1	1	3	3	1	2.33	2.33
21	*Machilus nanmu*	3	1	3	3	1	2.33	7
22	*Lindera megaphylla*	3	1	3	3	1	2.33	7
23	*Lindera communis*	2	1	3	3	1	2.33	4.67
24	*Platanus acerifolia*	2	3	2	2	1	1.67	10

25	*Grevillea robusta*	1	3	2	3	1	2	6
26	*Liquidambar formosana*	2	1	3	3	1	2.33	4.67
27	*Celtis sinensis*	1	1	3	3	3	3	3
28	*Morus alba*	2	2	3	3	3	3	12
29	*Morus cathayana*	2	2	2	2	3	2.33	9.33
30	*Broussonetia papyrifera*	2	3	2	3	3	2.67	16
31	*Pterocarya stenoptera*	1	3	3	3	3	3	9
32	*Castanea mollissima*	2	2	2	2	2	2	8
33	*Castanopsis fissa*	2	1	2	2	2	2	4
34	*Quercus acutissima*	3	3	3	2	1	2	18
35	*Quercus variabilis*	3	3	3	2	1	2	18
36	*Cyclobalanopsis glauca*	3	0	3	2	1	2	0
37	*Alnus cremastogyne*	1	3	3	2	3	2.67	8
38	*Casuarina equisetifolia*	2	3	3	2	2	2.33	14
39	*Camellia synaptica*	3	1	3	2	2	2.33	7
40	*Firmiana simplex*	3	2	3	2	1	2	12
41	*Brachychiton acerifolius*	1	1	3	2	1	2	2
42	*Reevesia pubescens*	2	1	3	2	1	2	4
43	*Salix babylonica*	2	3	3	2	1	2	12
44	*Diospyros lotus*	1	1	3	2	3	2.67	2.67
45	*Styrax japonicus*	1	1	3	2	1	2	2
46	*Sinojackia xylocarpa*	1	1	3	2	1	2	2
47	*Melliodendron xylocarpum*	1	1	3	2	1	2	2
48	*Chaenomeles cathayensis*	2	1	3	2	2	2.33	4.67
49	*Photinia serratifolia*	1	1	3	2	2	2.33	2.33
50	*Malus halliana*	2	1	3	2	2	2.33	4.67
51	*Pyrus ussuriensis*	1	1	3	1	2	2	2
52	*Prunus mume* f. *albo-plena'Zi Di Bai'*	2	1	3	2	2	2.33	4.67
53	*Prunus mume* f. *viridicalyx'Bian lv e'*	2	1	3	2	2	2.33	4.67
54	*Prunus mume* var. *bungo*	2	1	3	1	2	2	4

55	*Prunus × blireana‘Meiren’*	2	1	3	1	2	2	4
56	*Prunus campanulata*	3	1	3	2	2	2.33	7
57	*Prunus × subhirtella*	2	1	3	2	2	2.33	4.67
58	*Prunus campanulata‘Plena’*	2	1	3	2	2	2.33	4.67
59	*Prunus‘Canton’*	2	1	3	2	2	2.33	4.67
60	*Prunus serrulata*	2	1	3	2	1	2	4
61	*Prunus × yedoensis*	2	1	3	2	2	2.33	4.67
62	*Prunus cerasifera*	1	1	3	2	2	2.33	2.33
63	*Prunus pseudocerasus*	1	1	3	2	2	2.33	2.33
64	*Prunus cerasoides*	1	1	3	2	2	2.33	2.33
65	*Prunus persica‘Duplex’*	1	1	3	1	2	2	2
66	*Prunus persica*	2	1	3	2	2	2.33	4.67
67	*Acacia podalyriifolia*	3	1	3	2	3	2.67	8
68	*Acacia mearnsii*	2	1	3	3	3	3	6
69	*Albizia kalkora*	1	1	1	2	3	2	2
70	*Zenia insignis*	1	1	3	2	1	2	2
71	*Bauhinia variegata*	2	1	3	2	2	2.33	4.67
72	*Robinia pseudoacacia*	1	1	3	2	2	2.33	2.33
73	*Erythrina variegata*	1	0	3	2	1	2	0
74	*Erythrina corallodendron*	2	1	3	2	1	2	4
75	*Punica granatum*	2	1	3	2	3	2.67	5.33
76	*Lagerstroemia indica*	3	1	3	2	1	2	6
77	*Syzygium jambos*	3	1	2	2	1	1.67	5
78	*Syzygium nervosum*	1	1	3	2	1	2	2
79	*Callistemon viminalis*	3	1	2	2	1	1.67	5
80	*Melaleuca cajuputi* subsp. *cumingiana*	1	1	2	2	3	2.33	2.33
81	*Acca sellowiana*	3	1	2	2	1	1.67	5
82	*Davidia involucrata*	2	1	3	2	2	2.33	4.67
83	*Alangium chinense*	2	2	2	2	1	1.67	6.67
84	*Cornus wilsoniana*	1	1	3	2	3	2.67	2.67

85	*Cornus capitata*	2	1	3	2	3	2.67	5.33
86	*Cornus controversa*	2	1	3	2	1	2	4
87	*Euonymus maackii*	1	1	3	2	1	2	2
88	*Mallotus tenuifolius*	2	2	3	2	1	2	8
89	*Triadica sebifera*	2	1	3	2	2	2.33	4.67
90	*Bischofia javanica*	1	2	3	2	1	2	4
91	*Koelreuteria paniculata*	3	2	3	2	2	2.33	14
92	*Sapindus saponaria*	2	1	2	2	2	2	4
93	*Aesculus chinensis* var. *wilsonii*	2	0	3	2	1	2	0
94	*Acer catalpifolium*	1	1	3	2	1	2	2
95	*Acer palmatum*	1	1	3	2	1	2	2
96	*Acer buergerianum*	1	1	3	2	1	2	2
97	*Toxicodendron vernicifluum*	1	1	3	2	1	2	2
98	*Rhus potaninii*	1	0	3	1	2	2	0
99	*Choerospondias axillaris* var. *pubinervis*	3	1	3	2	1	2	6
100	*Melia azedarach*	1	2	2	3	1	2	4
101	*Chukrasia tabularis*	2	1	3	2	2	2.33	4.67
102	*Toona sinensis*	1	1	3	2	3	2.67	2.67
103	*Brassaiopsis glomerulata*	2	0	3	2	1	2	0
104	*Brugmansia arborea*	3	1	3	3	1	2.33	7
105	*Vitex negundo* var. *cannabifolia*	3	0	3	2	2	2.33	0
106	*Fraxinus rhynchophylla*	1	1	3	2	1	2	2
107	*Fontanesia phillyreoides subsp. fortunei*	1	1	2	2	1	1.67	1.67
108	*Ligustrum lucidum*	3	2	3	2	1	2	12
109	*Paulownia fortunei*	3	2	3	2	1	2	12
110	*Jacaranda mimosifolia*	1	1	2	2	3	2.33	2.33
111	*Trachycarpus fortunei*	1	2	2	2	1	1.67	3.33
112	*Livistona chinensis*	2	0	3	2	1	2	0

113	*Ehretia macrophylla*	2	0	3	2	1	2	0
114	*Crateva formosensis*	3	0	3	2	2	2.33	0
